# Identification of Natural Products as SENP2 Inhibitors for Targeted Therapy in Heart Failure

**DOI:** 10.3389/fphar.2022.817990

**Published:** 2022-04-01

**Authors:** Somayye Taghvaei, Farzaneh Sabouni, Zarrin Minuchehr

**Affiliations:** ^1^ Department of Medical Biotechnology, National Institute of Genetic Engineering and Biotechnology, Tehran, Iran; ^2^ Department of Systems Biotechnology, National Institute of Genetic Engineering and Biotechnology, Tehran, Iran

**Keywords:** SENP2, natural compounds, betanin, heart failure, molecular docking, molecular dynamics simulation

## Abstract

**Aims:** Sentrin-specific protease -2 (SENP2) is involved in deSUMOylation. Increased deSUMOylation in murine hearts by *SENP2* upregulation resulted in cardiac dysfunction and congenital heart defects. Natural compounds via regulating cell proliferation and survival, induce cell cycle cessation, cell death, apoptosis, and producing reactive oxygen species and various enzyme systems cause disease prevention. Then, natural compounds can be suitable inhibitors and since SENP2 is a protein involved in heart disease, so our aim was inhibition of SENP2 by natural products for heart disease treatment. Material and methods: Molecular docking and molecular dynamics simulation of natural products i.e. Gallic acid (GA), Caffeic acid (CA), Thymoquinone (TQ), Betanin, Betanidin, Fisetin, and Ebselen were done to evaluate the SENP2 inhibitory effect of these natural products. The toxicity of compounds was also predicted. Results: The results showed that Betanin constituted a stable complex with SENP2 active site as it revealed low RMSD, high binding energy, and hydrogen bonds. Further, as compared to Ebselen, Betanin demonstrated low toxicity, formed a stable complex with SENP2 via four to seven hydrogen bonds, and constituted more stable MD plots. Therefore, depending upon the outcomes presented herein, Betanin significantly inhibited SENP2 and hence may be considered as a suitable natural compound for the treatment of heart failure. Further clinical trials must be conducted to validate its use as a potential SENP2 inhibitor.

## 1 Introduction

Heart failure after myocardial infarction is an increasing health obstacle worldwide (Virani et al., 2020) and is basically made by the low regenerative capacity of the adult human heart upon damage (Payan et al., 2020). Although new drugs and reperfusion therapy were developed, the unalterable loss of cardiomyocytes led to cardiac remodeling and, next, heart failure (Ongstad and Gourdie, 2016). Therefore, the identification of new targets to increase cardiac regeneration is a promising strategy to revert the development of heart failure after myocardial infarction. Small Ubiquitin-like Modifier (SUMO) modification is implicated in various cellular processes including protein trafficking, transcriptional regulation, protein stability, cell death, and survival (Seeler and Dejean, 2003). Various SUMOylated proteins preferably associate with particular complexes such as promyelocytic leukemia (PML) bodies and the nuclear pores (Melchior et al., 2003; Müller et al., 2004). SUMOylation (conjugation of SUMO) includes three steps: processing, conjugation, and transition. The transition process includes covalent conjugation of SUMO polypeptides to targets (Gill, 2004). SUMO modification plays a role in cardiac development and function (Wang and Schwartz, 2010; Wang, 2011). SUMO conjugates can be easily deconjugated by SENPs (sentrin-specific proteases) (Mukhopadhyay and Dasso, 2007). Cardiac transcription factors such as Nkx2.5, GATA4, and myocardin are SUMO targets that refer to the role of SUMOylation in cardiovascular development (Wang et al., 2004; Wang et al., 2007; Wang et al., 2008; Wang et al., 2011).

SENP2, a key member of the SENPs family, modulates embryonic development (Kang et al., 2010), fatty acid metabolism (Koo et al., 2015), atherosclerosis (Heo et al., 2015), and neurodegenerative diseases (Fu et al., 2014). This protein may be a SUMOylation suppressor. *SENP2* upregulation in MCF7 breast cancer cells was led to reduced glycolysis, but SENP2 knockout in MEF cells resulted in enhanced glycolysis (Tang et al., 2013). SENP2 prevents keratinocyte migration by targeting NDR1 for deSUMOylation and inhibiting wound healing (Xiao et al., 2019). *SUSP4* (mouse SENP2) overexpression repressed cell growth, but *SUSP4* knockdown by RNA interference (RNAi) increased cell growth (Lee et al., 2006). Like SENP1, SENP2 is also complicated in the organization of gene expression programs in developmental procedures. The promoters of SENP1 and SENP2 possess response elements (REs) that bind to specific transcription factors (TFs). Both SENP1 and SENP2 can impress their transcription by deSUMOylation of transcription factors (Hickey et al., 2012)**.**

The studies have also described a positive effect of SENP2 on the transcriptional activity of nuclear receptors such as androgen receptor (AR), progesterone receptor (PR), and ER-related receptor (ERR2) (Nait Achour et al., 2014). SUMOylation and deSUMOylation are reversible and dynamic processes which when interrupted result in abnormal organogenesis, i.e., development of cleft lip/palate (Alkuraya et al., 2006; Song et al., 2008).

Disturbed flow through SUMOylation of p53 and extracellular signal-regulated kinase 5 (ERK5) lead to atherosclerotic plaque constitution (Heo et al., 2013). Yong Kim *et al.* have been reported *SENP2* upregulation with enhancing deSUMOylation in murine hearts results in cardiac dysfunction and congenital heart defects but *SUM O 1* overexpression can improve cardiac structural formation in SENP2-Tg mice (Kim et al., 2012). Chen *et al.* also showed the loss of SENP2-mediated Akt deSUMOylation and increased Akt kinase activity decrease glycogen synthase kinase 3 beta (GSK3β) levels and subsequently promote cardiomyocyte proliferation and angiogenesis. SENP2 knockdown increased cardiomyocyte dedifferentiation and proliferation both *in vitro* and *in vivo*. SENP2 deficiency could also moderate cardiac remodeling and better cardiac function after myocardial infarction (Chen et al., 2021). After birth, overexpression of *SENP2* alters cardiomyocyte division and causes congenital heart defects and cardiac dysfunction (Kim et al., 2012). Then due to its significant role in the development of many diseases especially heart failures, SENP2 can be an attractive target for drug discovery.

The *in vivo* and *in vitro* studies also demonstrated which natural compounds, especially phytochemicals, minerals, and vitamins, prevent cancer. More than 3,000 plant species have been reported in modern medicine (Millimouno et al., 2014). Natural compounds have many anti-cancerous and anti-turmeric properties such as anti-oxidative antiangiogenic, antiproliferative, and apoptotic effects (Alemi et al., 2013; Rashid et al., 2019; Ahmadi et al., 2020). Toona sinensis (leaf extract) had anti-cancer effects on prostate cancer cells and also caused apoptosis (Chen et al., 2009).Chin Leow *et al.* reported that curcumin has great therapeutic potential for the treatment of osteosarcoma (Leow et al., 2010). Curcumin has been reported to control autophagy, leading to the inhibition of several types of cancer cell proliferation (for example, chronic myeloid leukemia, malignant glioma, and oesophageal cancer cells) (Jia et al., 2009; Aoki et al., 2007; O'Sullivan-Coyne et al., 2009). Oridonin has been found to show significant anti-proliferative activity, especially inhibiting tumor growth, thus resulting in cancer cell death of melanoma and cervical carcinoma cells (Abelson, 1990; Cui et al., 2006).

Since enhanced deSUMOylation in murine hearts through overexpression of *SENP2* led to congenital heart defects and cardiac dysfunction (Kim et al., 2012), we require inhibitor(s) which efficiently bind to SENP2, inhibit SENP2, and are used for heart failure treatment. Among the treatment methods for heart diseases, we can name Cardiac Hospitalization Atherosclerosis Management Program (CHAMP) (Fonarow et al., 2001) and cell therapy (Wollert and Drexler, 2010). Natural products are inexpensive with lower side effects compared with these treatment methods.

According to our previous studies on these secondary metabolites and observation of their anti-inflammatory and antioxidant effects in various cancer cell lines and the nervous system cells on SENP1 protein (Amiraslani et al., 2012; Alemi et al., 2013; Esmaeilzadeh et al., 2013; Rashid et al., 2019; Ahmadi et al., 2020; Taghvaei et al., 2021a; Taghvaei et al., 2021b) and considering that SENP2 protein is one of the factors involved in heart diseases, we suggested these compounds may also affect SENP2 and inhibition of SENP2 can be performed by these compounds to heart failure treatment (Kim et al., 2012; Chen et al., 2021).

Then, our aim was the selection of compounds with more affinity, among natural compounds, for decreasing *SENP2* expression for the treatment of heart disease. Our study suggests natural products can be applied as starting points in the development of highly potent SENP2 inhibitors for therapeutic and biological targets. For this purpose, molecular docking was used for the measurement of affinity of natural products including Gallic acid (GA), Caffeic acid (CA), Thymoquinone (TQ), Betanin, Betanidin, Fisetin, and Ebselen as a control (Bernstock et al., 2018) to SENP2. Molecular dynamics (MD) simulation was also used to verify molecular docking and essential dynamic analysis was applied to more surveys. The toxicity of compounds was also predicted. Computational tools, such as computational ADME/Tox properties, ligand-based VS., and MD have extreme importance in pharmaceutical research and industry, to select molecules with therapeutic potential (Ciemny et al., 2018). This is an *in silico* study to inhibit SENP2 by natural products for use in heart failure treatment. By providing inhibition of SENP2, cardiac function can be improved. This study also introduces SENP2 as a therapeutic target.

## 2 Material and Methods

In this process, we selected a suitable protein structure based on the resolution and the amino acids of the binding site. In order to the binding of the chemical compounds to SENP2 after the determination of the active site, molecular docking was carried out. The compounds were also molecular docked by Lipinski’s Rule of Five and various modes consisting of ADMET (Hodgson, 2001) and TOPCAT (Taylor, 2005). Then, in order to find a compound bonded with higher efficiency to the active site of SENP2, the MD simulation and essential dynamic analysis were applied. Lastly, the toxicity of compounds was predicted.

### 2.1 Molecular Docking

#### 2.1.1 Protein and Ligand Preparation

PDB ID: 1TH0 for SENP2 study was extracted from the RCSB PDB database (https://www.rcsb.org) (Rose et al., 2016). The ligand structure was also obtained through http://zinc.docking.org/(Irwin et al., 2012). Before initiating docking, protein and ligand structure were prepared.

Molecular docking of SENP2 was performed with the compounds GA, CA, TQ, Betanin, Betanidin, Fisetin, and Ebselen as control compounds (Supplementary Table S1) using AutoDock4 software (Morris et al., 2009). Molecular docking in the position: x center = -11.518 -y center = 15.863 -z center = 82.218 was performed by considering amino acids of the active site consisting of Leu411, Asn412, His474, Lys476, Val477, His478, Trp479, Met497, Gln542, Trp410Gly545, Ser546, Asp547, Ser548, and Gly549 which were used by other researches (Kumar et al., 2014). Active site verification was performed by the PockDrug server (Hussein et al., 2015).

Molecular docking of the ligands with SENP2 was done by AutoDock4 software packages (Morris et al., 2009). Polar hydrogen atoms were incorporated, non-polar hydrogens were merged, and Gasteiger charges were added. The docking operation was fulfilled in a grid box consisting of 60 × 60 × 60 (x, y, z) points at the center with 100 runs and the grid resolution of 0.375 Å to cover the SENP2 active site. Other parameters were set to default amounts (Taghvaei et al., 2021a; Taghvaei et al., 2021b). The best pose was selected for MD simulation. LigPlot software was used to analyze the docking and MD simulation results obtained from AutoDock4.

### 2.2 Molecular Dynamics Simulation

#### 2.2.1 Molecular Dynamics Simulation and Binding Free Energy Prediction

PRODRG (Schüttelkopf and Van Aalten, 2004) was applied for topology generation of ligand for the GROMOS force field. MD simulation of docking complexes was done by the genuine union tool of GROMACS (Van Der Spoel et al., 2005). In the MD simulation process, each one of the complexes immersed in a dodecahedron-modeled box (x, y, and z) with 238.58 nm^3^ and with 1nm of space between the protein periphery and the box edges. We used SPC/E water molecules in order to solvate the system. System neutralization was applied by the addition of five chloride ions for all compounds, Ebselen, and free-SENP2. To instability prevention in MD simulation, the solvated system was subjected to 1,000 cycles minimization. All MD simulations were performed by the GROMACS 4.6.5 package (Abraham et al., 2014) by the GROMOS53a6 force field (Oostenbrink et al., 2005). Before the MD simulation run, the temperature of the crystal structure was attained to 300 K and then equilibrated during 100 ps at the conditions of constant volume and temperature (NVT). Afterward, the system was altered to the constant pressure and temperature (NPT) and equilibrated for 100 ps. The non-bonded cut-off was set at 10 Å and for every 5 steps, the non-bonded pair list has been updated. All MD simulations were performed with the PME parallel version (Guckel, 1999) in the GROMACS suit. LINKS mode was used to constrain all hydrogen bonds and motion equation integration (Essmann et al., 1995) and structural snapshots were flushed every 500 steps (Van Der Spoel et al., 2005). A 50 ns MD simulation of SENP2 in 25 ×10^6^ steps alone (free-SENP2), and in the presence of natural compounds were carried out as mentioned. The computation and analysis of the average hydrogen bonds number between receptor and ligand were performed by g_hbond. The cutoff radius between the acceptor and the donor was 0.35 nm (Taghvaei et al., 2021b).

#### 2.2.2 Molecular Mechanics–Poisson Boltzmann Surface Area

MM-PBSA estimates the free energies and the scoring function in the computational drug design which is excessively applied to the survey of bio-molecular interactions (Kumari et al., 2014; Taghvaei et al., 2021a). The MM-PBSA mode was made in the GROMACS plan was used to compute the difference of the free energies (ΔG) among ligand configurations and free-SENP2.

#### 2.2.3 Data Analysis and Visualization Software

Our 2D images were created using Discovery Studio (Studio, 2008) and 3D images were made using PyMOL (Seeliger and de Groot, 2010).

#### 2.2.4 Analysis of Molecular Dynamics Trajectories

The generated results were analyzed using, g_rms, g_rmsf, g_gyrate, g_sasa, g_hbond, g_mindist, and do_dssp, etc. All plots and figures were prepared using GRACE software (http://plasma-gate.weizmann.ac.il/Grace/).

### 2.3 Essential Dynamics

Essential dynamics, known as Principal Component Analysis (PCA), can show the collective atomic motion of free-SENP2, GA, CA, TQ, Betanin, Betanidin, Ebselen, and Fisetin by the GROMACS tool. The principal component analysis was computed using g_covar and g_anaeig built-in functions of the GROMACS package. PCA is a standard protocol for the characterization of eigenvectors and the projection across the first PC1 and PC2 (Amadei et al., 1993; Udhaya Kumar et al., 2020; Taghvaei et al., 2021c).

### 2.4 Investigation of Toxicity by in Silico Method

After molecular docking, MD simulation, and essential dynamic analysis were done. It is necessary to investigate the toxicity of these compounds. Because when a chemical compound is used as an oral drug, it first enters the stomach. The drug must be resistant to the vicinity of gastric acid and must be able to enter intestinal cells after passing through the stomach. The drug must be able to enter the bloodstream through the intestinal wall and go through the blood vessels to the liver. In the liver, a drug must show resistance to metabolism, until it eventually enters the bloodstream and reaches its target. Thus, the drug that both computational and laboratory effects had verified as best on the target protein cannot be used as an oral drug, because it may be changed during this complex route and may not actively achieve its goal. On the other hand, about 20 years ago, before the introduction of computational methods, about 50% of potential therapeutic compounds failed before entering the clinical stage. As a result, a successful drug is not necessarily the best inhibitor of its target, because a drug can be introduced as a successful drug which has the necessary criteria from absorption, distribution, metabolism, excretion, and toxicity (ADMET). Knowing these can increase the speed of drug design.

The process of designing and building a potential therapeutic combination is very costly and time-consuming. As a result, the use of different computational methods to reduce the failure of these candidate compounds is very important and the use of ADMET methods has reduced the failure rate to less than 8% (Merlot, 2010; Cheng et al., 2012). Various software have been designed for this process. Some isfree such as Toxtree, SARpy, T.E.S.T, and CAESAR, and some are commercially available such as ADMET predictor, ACD/TOX suite, TOPKAT, and Derek Nexus. They each have their own advantages over the others, which makes their choice conditional on the type of study (Bakhtyari et al., 2013).

#### 2.4.1 Lipinski Rule of Five

Lipinski Rule of Five consists of molecular weight <500D, hydrogen bond acceptors less than 10, hydrogen bond donors less than 5, logP<5, and Molar refractivity between 40-130 (Lipinski, 2000).

#### 2.4.2 Absorption, Distribution, Metabolism, Excretion, and Toxicity

The human body is constantly exposed to several compounds over time. During the development, several defense barriers have been created to inactivate them. These defenses include the family of cytochrome isoenzymes (CYP) 450 present in the liver, the active return of drug compounds using P-gp (Permeability-glycoprotein), the blood-brain barrier, and the kidneys. Because the range of chemical compounds is so wide, it is almost impossible to examine each of them in the laboratory. Further investigation is assisted by QSAR and QSTR methods. The basis of these methods is based on the principle that compounds with similar structures will have similar functions. So, if we select several known compounds with specific properties as a training series, we can predict the properties of similar compounds with unknown properties (Cheng et al., 2012). In the ADMET study according to the training series, a series of properties of compounds obtained, and the properties of new compounds are measured based on them.

#### 2.4.3 TOPKAT

As explained, the ADMET method is based on a training series. But there is a problem: that the small size of the series which can make the results somewhat erroneous. To solve this problem, a database called NTP (The National Toxicology Program) was introduced in which the effects of different chemical compounds at different concentrations were measured *in vitro* for a period of 2 years on mice and rats. The TOPKAT model is a tool for using this data as training series, using QSTR methods, and finding similar structures in the newly introduced structure to predict the toxic properties of new compounds. Because these training series contain a larger number of studied compounds that have been examined on living organisms for at least 2 years then, TOPCAT has more reliable results than ADMET. In the case of common indicators that are referred to in both ADMET and TOPKAT methods, the decision criteria are based on TOPKAT results.

## 3 Results

### 3.1 Molecular Docking Study

Molecular docking results demonstrate an exact and preferred orientation of natural products in the active pocket of the protein (Naqvi et al., 2018). The active site used was verified with a drug probability of 62%. The lowest binding free energy was computed with AutoDock4 and the compounds according to lowest binding energy included CA, GA, Betanin, Fisetin, Ebselen, Betanidin, and TQ, as shown in Table 1. CA, GA, Betanin, and Fisetin had better binding free energy than Ebselen. Energy items were the torsional and internal energy of the ligand, hydrogen bonds, intermolecular forces, electrostatic energy, van der Waals energy, and desolvation energy. Compounds bound deeper into the active pocket and perhaps diminished the accessibility of SENP2 which may be responsible for controlling its biological function.

**TABLE 1:** Molecular docking analysis results of natural compounds against SENP2 protein.

Natural compound	Lowest binding free energy (kcal/mol)
GA	−6.43
CA	−7.18
TQ	−4.42
Betanin	−5.53
Betanidin	−4.45
Fisetin	−4.96
Ebselen	−4.77

Hydrogen bonds and hydrophobic bonds between compounds and SENP2 were displayed in Table 2. LigPlot images were also displayed in Supplementary Figure S1. We observed GA and Betanidin constituted the most hydrogen bonds with the active site of SENP2. Ebselen and CA were also showed the most hydrophobic bonds with the active site of SENP2, as shown by Table 2.

**TABLE 2:** Amino acid residues contributed in molecular docking including hydrogen bond and hydrophobic bond.

Compound name	Hydrogen bond residues	Hydrophobic bond residues
GA	Val477, Leu411, Trp479, His474	Asn412, Trp410
CA	Leu411, Lys476	Asn412, His474, Trp479, Trp410, Val477
TQ	Trp479, Leu411	Thr404, Trp410, Lys476, Val477, Asn412, His474
Betanin	Asp401, Asn412	Glu414, Asp413, His474, Val477, Lys476
Betanidin	His474, Asp413, Asn412, Lys476	Val477, Trp410
Fisetin	Trp410, Asp413	His409, Thr404, Val477, Leu411, His474, Asn412
Ebselen	Gln499	Trp410, Arg475, Val477, His478, Met497, Gln542, Asn544, Gly545, Cyt548

### 3.2 Molecular Dynamics Simulation Method

MD simulation is a strong computational procedure to display the flexibilities of molecules (Syed et al., 2018). Structural information of the binding mechanism of natural products with the SENP2 was obtained through MD simulation.

#### 3.2.1 Free Energy Calculation

The binding energy for GA, CA, TQ, Betanin, Betanidin, Ebselen, and Fisetin were -179. 57, -195.599, -4.247, -328.872, -465.85, -72.844, and -89.984 kJ/mol which Betanin and Betanidin showed stronger binding compared with Ebselen, Table 3.

**TABLE 3:** Binding energy of MD simulation for compounds: GA, CA, TQ, Betanin, Betanidin, Ebselen, and Fisetin.

Compound name	Free binding energy (kJ/mol)
Betanidin	−465.85
Betanin	−328.872
CA	−195.599
GA	−179. 57
Ebselen	−89.984
Fisetin	−72.844
TQ	−4.247

#### 3.2.2 Average Potential Energy

The average potential energy was monitored to determine the equilibration of the systems before the MD analysis. At a constant temperature (300 K), the average potential energy for free-SENP2 was estimated to be -647,631 kJ/mol. Average potential energy for GA, CA, TQ, Betanin, Betanidin, Ebselen, and Fisetin, were -642,154 kJ/mol, -655,213 kJ/mol, -646,049 kJ/mol, -656,986 kJ/mol, 656,179 kJ/mol, -653,727 kJ/mol, and -654,855 kJ/mol, respectively, Table 4. The average potential energy of Betanin and Betanidin were better than others even than Ebselen.

**TABLE 4:** Potential energy of natural compounds, free-SENP2, and Ebselen.

Compound name	Potential energy (kJ/mol)
Betanin	−656,986
Betanidin	656,179
CA	−655,213
Ebselen	654,855
Fisetin	−653,727
Free-SENP2	−647,631
TQ	−646,049
GA	−642,154

#### 3.2.3 Structural Deviations and Compactness

**FIGURE 1:**
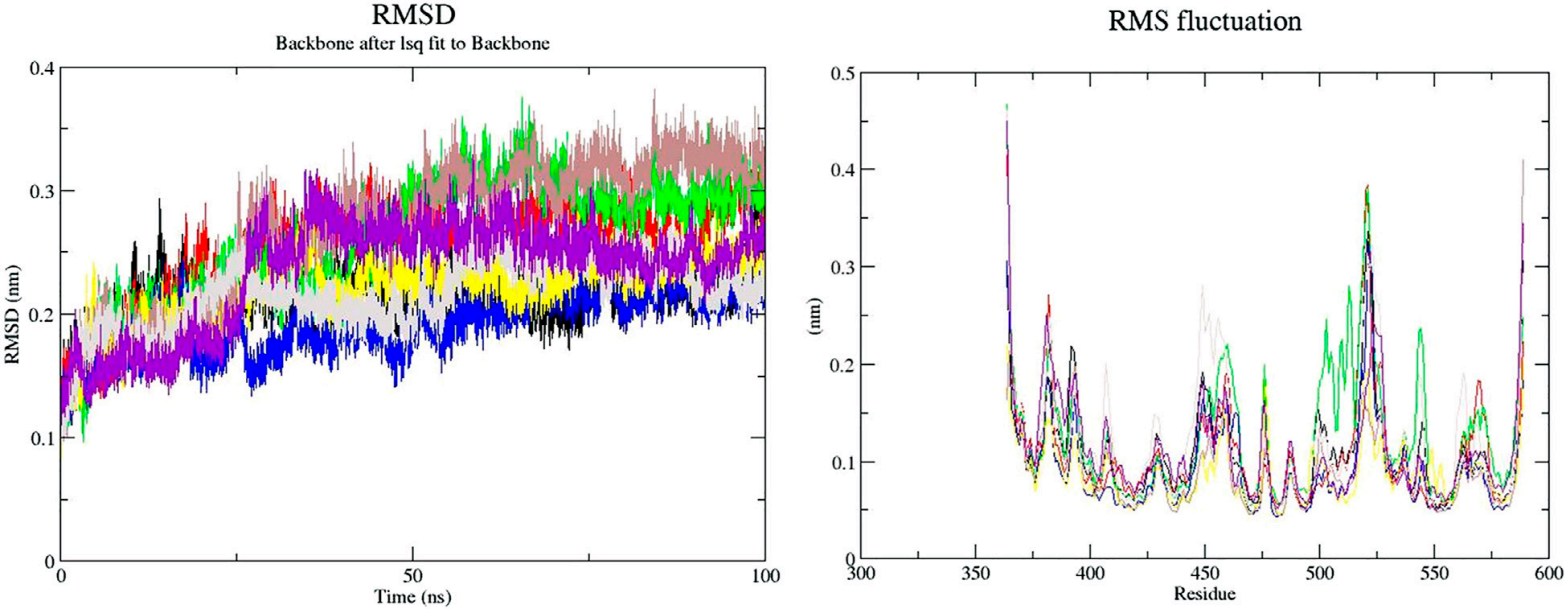
RMSD and RMSF plots of free-SENP2 and SENP2 complexes (black) free-SENP2, red) GA, green) CA, blue) TQ, yellow) Betanin, brown) Betanidin, gray) Ebselen, and purpule) Fisetin).

The binding of a ligand to the protein can generally induce significant conformational alterations in the structure. The root mean square deviation (RMSD) parameter was computed to observe whether the structure of a protein is stable and near the experimental structure (Taghvaei et al., 2021a). The average RMSD value for free-SENP2, GA, CA, TQ, Betanin, Betanidin, Ebselen, and Fisetin were found to be 0.21, 0.23, 0.24, 0.17, 0.21, 0.23, 0.20, and 0.21 nm, respectively. We observed TQ, Fisetin, and Betanin have lower RMSD than others and are very close to free-SENP2 and Ebselen. The RMSD plot proposed that the binding of all these compounds were significantly stabilized the structure of SENP2 and were resulted in fewer structural deviations from its native conformation and were equilibrated throughout the 50 ns MD simulation (Figure 1).

To compute the average fluctuation of residues, the root mean square fluctuation (RMSF) of the SENP2 upon ligands binding was drawn as a function of residue number (Figure 1). The RMSF plot demonstrated residual fluctuations present at various regions of SENP2, the especially active sites. We observed Betanidin to have lower fluctuations.

**FIGURE 2:**
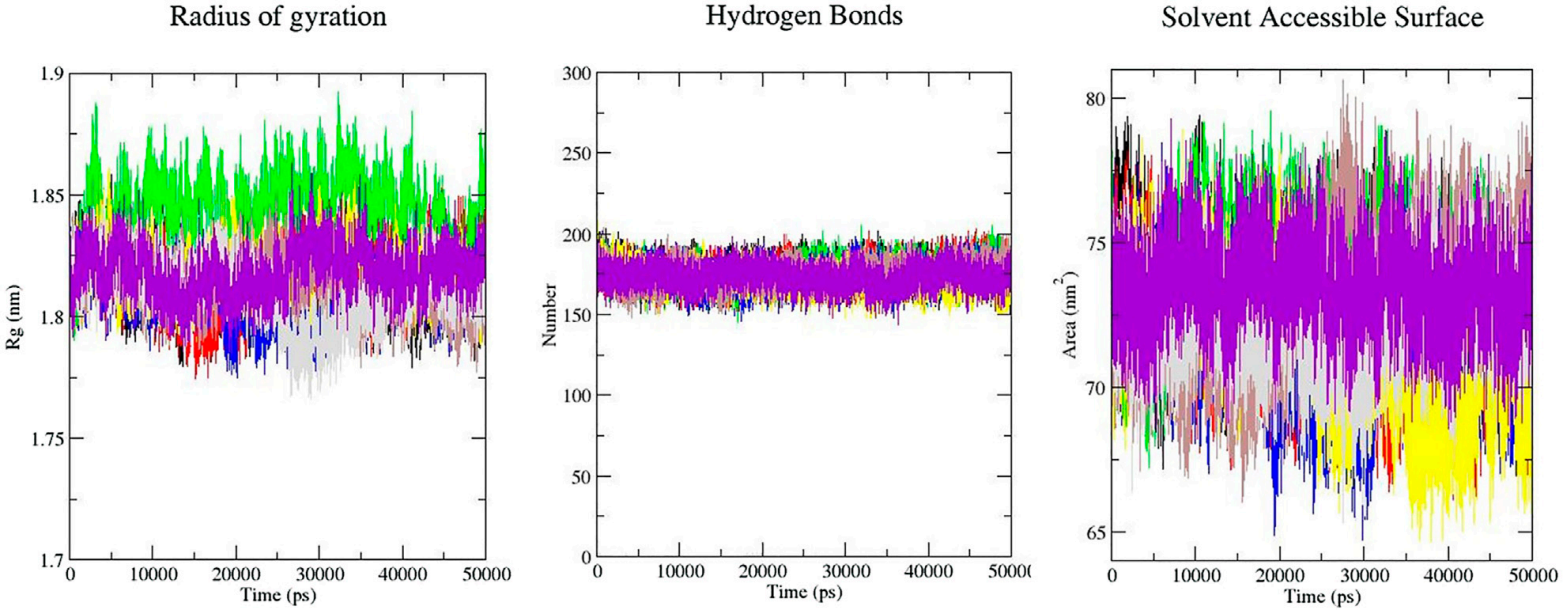
Rg, intramolecular hydrogen bonds, and SASA plots of SENP2 complexes, black) free-SENP2, red) GA, green) CA, blue) TQ, yellow) Betanin, brown) Betanidin, gray) Ebselen, and purple) Fisetin.

*Rg* is an indicator of the level of structure compaction, i.e. the polypeptide is unfolded or folded (Taghvaei et al., 2021b; Taghvaei et al., 2021c). The radius of gyration (*Rg*) is a parameter that is usually calculated to gain insights into the stability of the protein in terms of alteration in the volume of protein. A protein with higher *R*g values has a flexible packing. The average *Rg* values for free-SENP2, GA, CA, TQ, Betanin, Betanidin, Ebselen, and Fisetin were found to be 1.81, 1.82, 1.84, 1.81, 1.82, 1.81, 1.81, and 1.82 nm, respectively (Figure 2). These differences in the *Rg* values were not remarkable and SENP2-compounds complexes were stable.

#### 3.2.4 Solvent Accessible Surface Area

Calculation of SASA (Solvent Accessible Surface Area) supplies the conformational changes in protein upon ligand binding. Estimation of SASA provides information about the conformational changes in protein upon ligand binding (Taghvaei et al., 2021b). The average SASA values for free-SENP2 and SENP2-compounds were also monitored in 50 ns MD simulations. The average SASA values for free-SENP2, GA, CA, TQ, Betanin, Betanidin, Ebselen, and Fisetin were found to be 73, 72, 74, 72, 71, 73, 72, and 74 nm^2^, respectively. There were negligible differences, (Figure 2).

#### 3.2.5 Hydrogen Bonds Analysis

The molecular identification between a protein and ligand is related to the hydrogen bonding pattern which supplies a specificity and directionality of interaction (Taghvaei et al., 2021a). We have computed hydrogen bonds pairing (within 0.35 nm) between SENP2 and ligands in the solvent condition. An average number of intramolecular hydrogen bonds were measured. We observed that the average number of intramolecular hydrogen bonds of free-SENP2, GA, CA, TQ, Betanin, Betanidin, Ebselen, and Fisetin were 177, 176, 176, 174, 172, 175, 174, and 174 hydrogen bonds, respectively, (Figure 2). We observed GA, CA, and Thymoquinone have more hydrogen bonds and potent binding. Of course, the differences were not significant.

**FIGURE 3:**
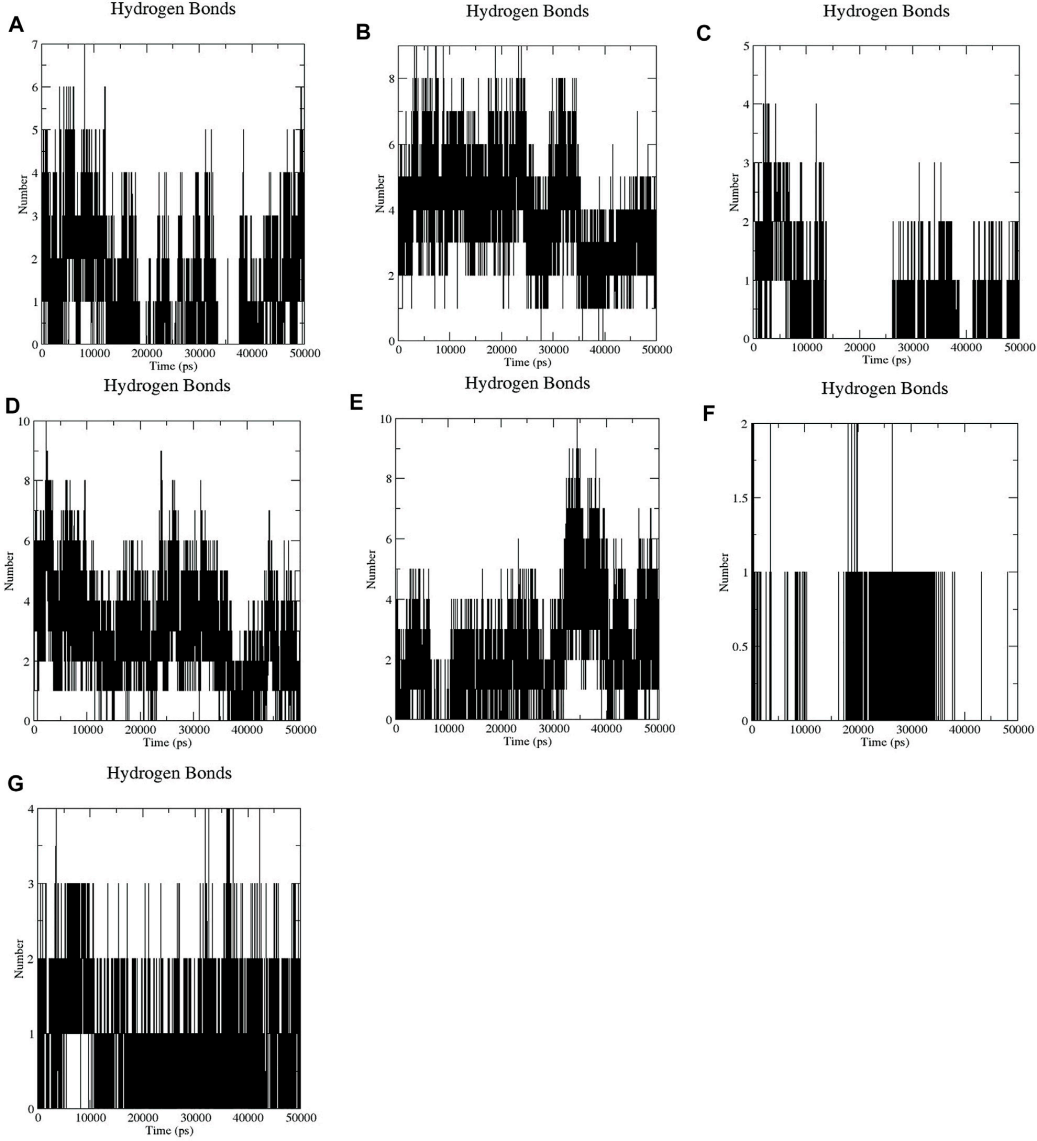
Intermolecular hydrogen bond plots of compounds **(A)** GA, **(B)** CA, **(C)** TQ, **(D)** Betanin, **(E)** Betanidin, **(F)** Ebselen, and **(G)** Fisetin.

It has been found that GA, CA, TQ, Betanin, Betanidin, Ebselen, and Fisetin bind to the active pocket of SENP2 with 4-5, 4–8, 2–3, 4–7, 4–7, 1, and two to three intermolecular hydrogen bonds, respectively (Figure 3).

#### 3.2.6 Distance Between SENP2 and Compounds

**FIGURE 4:**
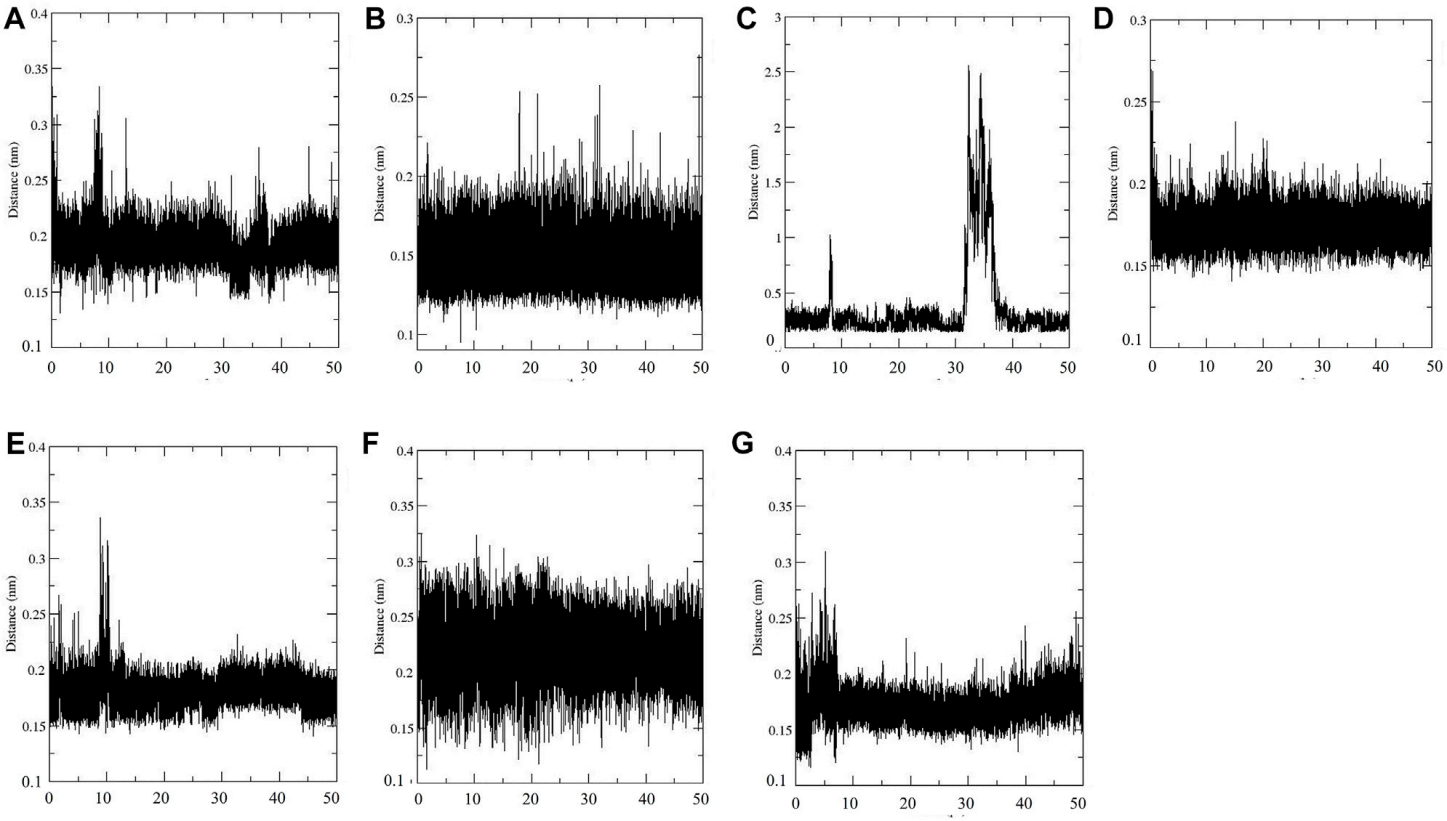
Min distance between SENP2 and compounds **(A)** GA, **(B)** CA, **(C)** TQ, **(D)** Betanin, **(E)** Betanidin, **(F)** Ebselen, and **(G)** Fisetin.

Distance between SENP2 and ligands were obtained using the embedded packages within GROMACS. Distance between GA, CA, TQ, Betanin, Betanidin, Ebselen, Fisetin and SENP2 were 0.19, 0.15, 0.36, 0.17, 0.18, 0.22, and 0.18 nm, respectively, see Figure 4. CA and Betanin represented the lowest distance with SENP2 protein.

#### 3.2.7 Secondary Structure Changes

**FIGURE 5:**
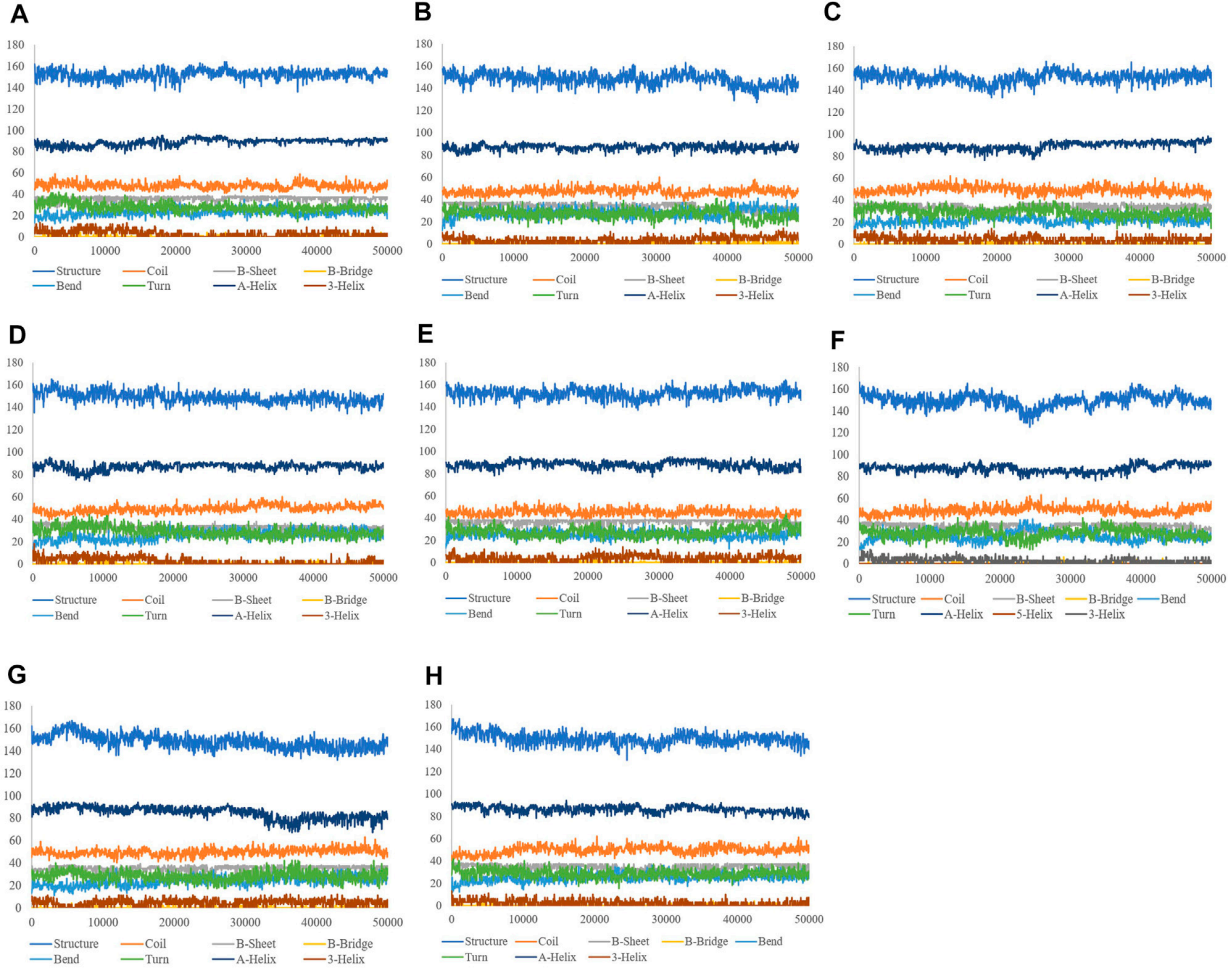
Secondary structure plots of compounds **(A)** GA, **(B)** CA, **(C)** TQ, **(D)** Betanin, **(E)** Betanidin, **(F)** Ebselen, **(G)** Fisetin, and **(H)** free-SENP2.

The aim of this analysis is the measurement of the alteration in the secondary structure of SENP2 upon binding to the compounds as a function of time. (Table 5). We did not observe any changes for Betanin. After Betanin, Ebselen showed the lowest changes including a decrease in bend and an increase in α-helix. Secondary structure changes for TQ included a decrease in bend, and an increase in α-helix and 3-helix. The most changes occurred in CA, GA, and Betanidin. Secondary structure changes for Fisetin also included an increase in β-sheet and 3-helix and a decrease in turn and α-helix. Although minor changes were seen in the secondary structure, the Betanin did not show any alteration (Figure 5).

**TABLE 5:** Secondary structure changes upon ligands binding.

DSSP	Structure	Coil	β-Sheet	β-bridge	Bend	Turn	α-helix	3-Helix
GA	0.67	0.21	0.16	0.00	0.1	0.12	0.39	0.01
CA	0.65	0.21	0.15	0.00	0.12	0.12	0.38	0.02
TQ	0.67	0.22	0.15	0.00	0.1	0.13	0.39	0.02
Betanin	0.66	0.22	0.15	0.00	0.11	0.13	0.38	0.01
Betanidin	0.67	0.2	0.16	0.00	0.11	0.12	0.39	0.02
Fisetin	0.66	0.22	0.16	0.00	0.11	0.12	0.37	0.02
Ebselen	0.66	0.22	0.15	0.00	0.11	0.12	0.39	0.01
Free-SENP2	0.66	0.22	0.15	0.00	0.11	0.13	0.38	0.01

#### 3.2.8 Data Visualization

**FIGURE 6:**
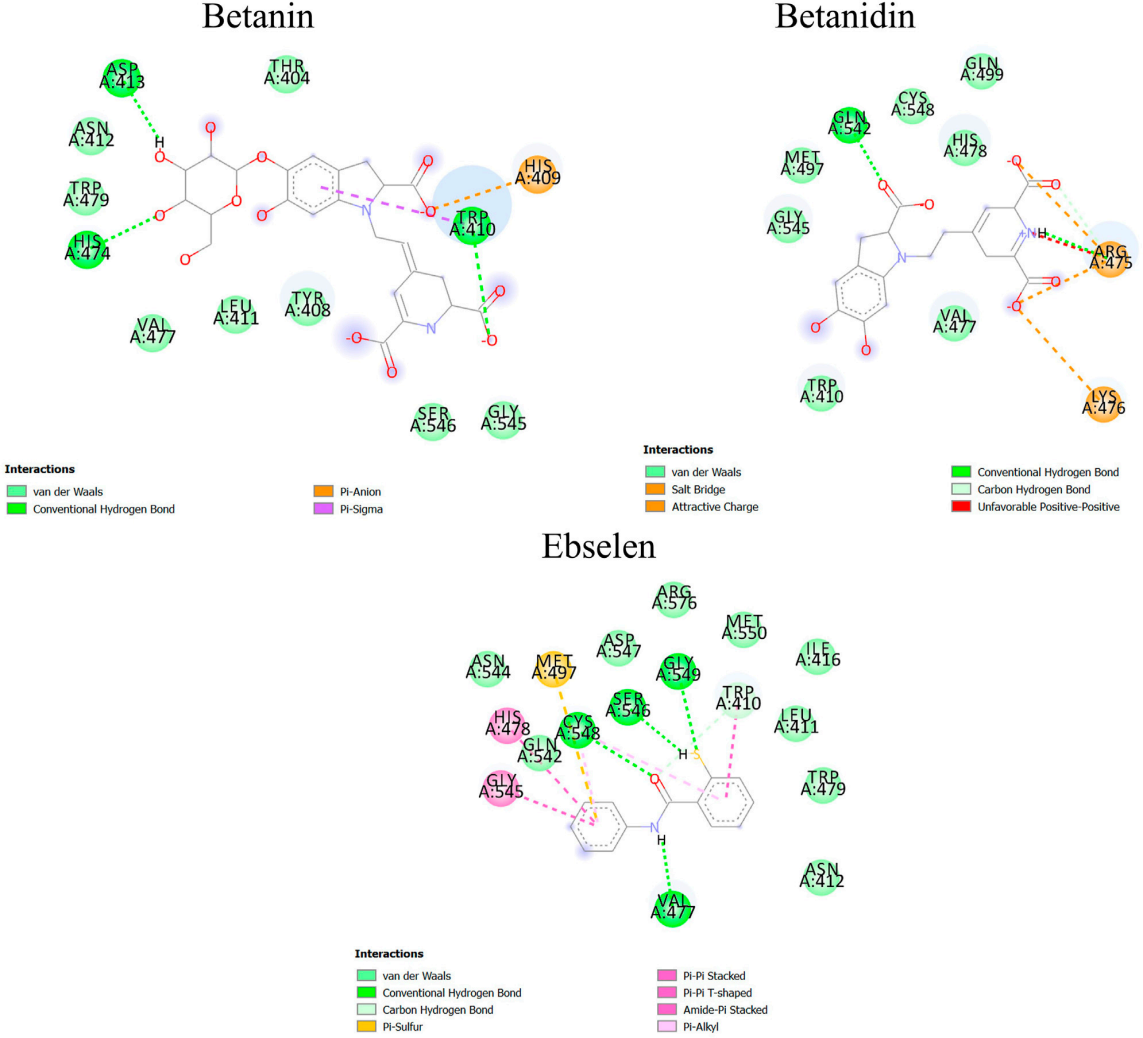
Binding between Betanin, Betanidin, and Ebselen with SENP2 by Discovery Studio after molecular dynamics simulation (2D structures).

**FIGURE 7:**
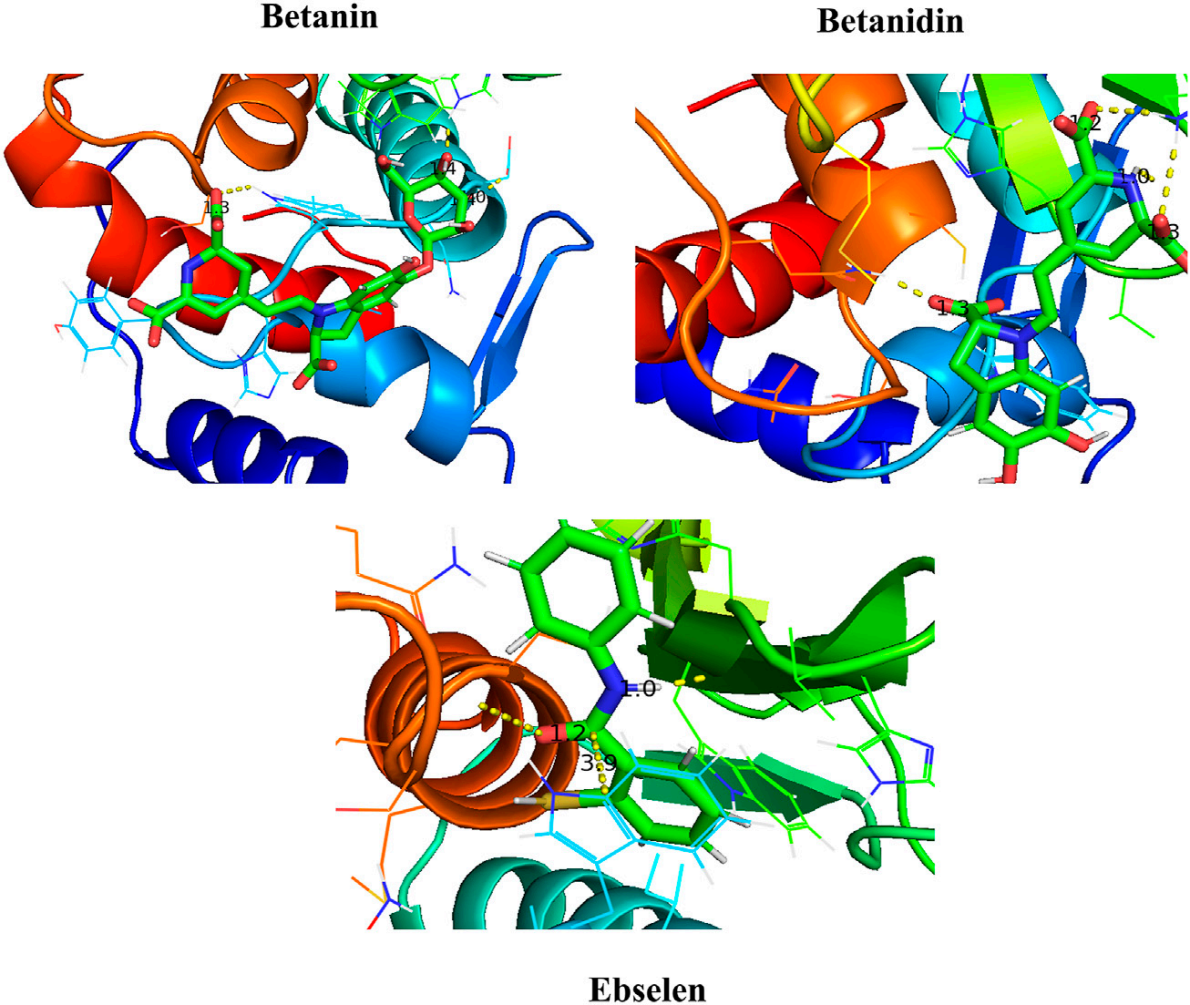
Binding between Betanin, Betanidin, and Ebselen with SENP2 by PyMOL after molecular dynamics simulation (3D structures).

After molecular dynamics simulation, the interaction between SENP2 and ligands by LigPlot. As we were displayed in Figure 5, Betanin constitutes bond with Thr404, Trp408, His409, Trp410, Leu411, Asn412, His474, Val477, Trp479, Gly545, and Ser546. Betanidin constitutes a bond with Trp410, Arg475, Lys476, Val477, His478, Met497, Gln499, Gln542, Gly545, and Ser548. Ebselen also binds to Trp410, Leu411, Asn412, Ile416, Val477, His478, Trp479, Met497, Gln542, Gly545, Ser546, Asp547, Ser548, and Gly549, Met550, Asn544, and Arg576. The binding of these compounds is with the active site of SENP2, see Figure 6 and Supplementary Figure S2. The 3D structures have also been displayed in Figure 7.

### 3.3 Essential Dynamics

**FIGURE 8:**
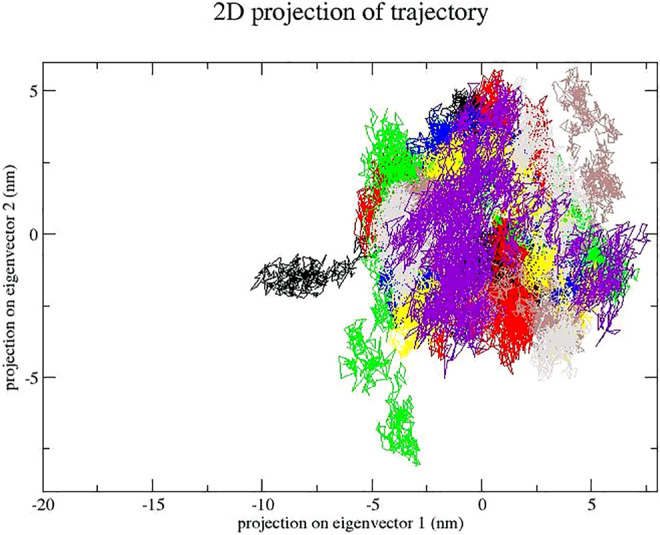
Principal component analysis. Projection of the motion for compounds (red) GA, green) CA, blue) TQ, yellow) Betanin, brown) Betanidin, gray) Ebselen, purple) Fisetin, and black) free-SENP2. in phase space along the PC1 and PC2.

The dynamics of GA, CA, TQ, Betanin, Betanidin, Ebselen, and Fisetin were obtained through a principal component analysis (PCA) (Kumar et al., 2019). The projection of trajectories of GA, CA, TQ, Betanin, Betanidin, Ebselen, and Fisetin during the molecular dynamic’s simulation in the phase space along the first two principal components (PC1, PC2) at 300 K were plotted in Figure 7. It predicts the large-scale collective motions for GA, CA, TQ, Betanin, Betanidin, Ebselen, and Fisetin. PCA analysis showed that these compounds change the structural dynamics of SENP2. Figure 8 plot clearly shows that TQ, Betanin, Ebselen, and Fisetin had fewer movements and occupied less space in phase space while free-SENP2 occupied more space which verifies the overall increased stability of TQ, Betanin, Ebselen, and Fisetin in the binding to SENP2. It is comparable to compounds MB_241, MB_250, and MB_266 in the study of Ahmad *et al.* for SARS-COV-2 inhibition (Ahamad et al., 2021). The PCA analysis results agreed with the results from MDS.

### 3.4 Investigation of Toxicity by in Silico Method

Druglikness is a qualitative meaning used in the design of a drug indicating how a substance is “druglike”. The drug-likeness properties of these compounds were gained using Lipinski’s Rule of Five, admetSAR, and TOPKAT. All of the compounds obeyed Lipinski’s Rule of Five. The results of the drug-likeness by ADMET and TOPKAT are presented in Supplementary Table S2, S3. The chemical properties of the identified compounds require to the determination of pharmacokinetic properties evaluated in terms of absorption, distribution, metabolism (how they interact with cytochromes), excretion (excretion of the kidney), and toxicity. AMES carcinogenicity was safe for all of the compounds and they were not a carcinogen and not a mutagen. The results of ADMET also showed TQ, Ebselen, and Fisetin able to cross the blood-brain barrier and the intestinal wall. So, these can be used for brain tumors and can be used orally. All the compounds were permeable to CaCO2 except Betanin and Betanidin. All compounds could be localized in the mitochondria except CA which could be localized in the nucleus. Only GA and CA cross the intestinal wall, but Betanin and Betanidin did not cross the intestinal wall and blood-brain barrier.

Other indicators that were evaluated at this stage were the ability to bind and suppress glycoproteins which are actively involved in the removal of xenobiotics from the cell. The ideal druglike compounds are compounds that do not bind to glycoproteins and therefore do not leave the cell. In this case, GA, CA, TQ, and Ebselen were not substrates for glycoproteins, but Betanidin, Betanin, and Fisetin were substrates for glycoproteins. The point to be considered is that from these selected compounds, ideal ones are neither glycoprotein substrates nor inhibitors. Because, these glycoproteins have other roles that by inhibiting them, these roles can be inhibited and the normal function of the cell is likely disturbed. Thus, GA, Betanidin, CA, Fisetin, Betanin, and Ebselen were not glycoprotein inhibitors, but TQ inhibits P-glycoprotein. All compounds were not an inhibitor of P-glycoprotein. Another indicator that has been measured is the possibility of metabolizing by cytochrome 450 and inhibiting this complex of metabolic proteins. The compound which cannot be metabolized can accumulate in the body and can lead to unwanted side effects. The compounds which can be metabolized by these proteins were selected. GA, CA, Fisetin, and TQ were not CYP450 substrates but Betanidin, Betanin, and Ebselen were CYP450 3A4 substrates. On the other hand, GA, TQ, CA, Betanidin, and Betanin were not inhibitors of CYP450, but Fisetin was an inhibitor of CYP450 1A2, and Ebselen was an inhibitor of CYP450 1A2, CYP450 2C9, CYP450 2D6, CYP450 2C19, and CYP450 3A4. Fish Toxicity (FHMT), Tetrahymena Pyriformis Toxicity (TPT), and Honey Bee Toxicity (HBT) were high for all compounds while, Honey Bee Toxicity (HBT) was low for Betanin, Betanidin, and Ebselen. Also, all the compounds represented weak inhibition potential of the human ether-a-go-go-related gene (hERG), which its expression has a significant role in the repolarization of the cardiac action potential (Sanguinetti et al., 1995).

The ADMET method is based on QSAR and QSTR methods, the number of properties proposed through statistical calculations, and the similarity between the studied compounds and compounds with approved properties. While in TOPKAT method, calculations are based on laboratory examination of a series of basic compounds in 2 years that their similar structures are used in other chemical structures. The results of these experiments predict the similar structures of the structure under study. As a result, differences in the studies between the two methods can be expected and, in such cases, a conclusion is made based on the TOPKAT results.

On the other hand, to evaluate the toxicity of the identified compounds based on the QSTR model, TOPKAT (Discovery Studio 2.5, Biovia, San Diego, CA, United States) was applied. This model is based on repetitive statistical methods with high credit ratings and is highly developed. In this model, the toxic effects of these compounds based on their chemical structure are predicted. The numeric values of TOPKAT software are divided into two categories. The first group of numbers contains from 0.0 to 1.0. These numbers are related to endpoint investigations which represent the probable calculated values for each of the compounds. The values from 0.0 to 0.3 represent the negative response of the compounds in the laboratory tests. While values between 0.7 and 1.0 represent a positive response in these experiments, the values between 0.3 and 0.7 indicate an intermediate state. The second group of numbers related to the amounts consumed by these compounds with a concentration value greater than 1.0. The results are presented in Supplementary Table S3. TOPKAT results showed all compounds are safe for the AMES mutagenicity test except Fisetin, CA, Betanin, and Ebselen. Also, in NTP Carcinogenicity tests, GA, CA, and Betanin in NTP Carcinogenicity Call (Male Rat) (v3.2) test were not carcinogenic, but Fisetin and Ebselen were carcinogenic. GA, Betanidin, TQ, and Betanin were not carcinogenic in NTP Carcinogenicity Call (Female Rat) (v3.2) test, but Fisetin and Ebselen were carcinogenic. GA, Betanidin, TQ, CA, Betanin, and Ebselen were not carcinogenic in NTP Carcinogenicity Call (Male Mouse) (v3.2) test, but Fisetin was carcinogenic. GA, TQ, CA, Fisetin, Betanin, and Ebselen were not carcinogenic in NTP Carcinogenicity Call (Female Mouse) test (v3.2) but Betanidin was carcinogenic. FDA Carcinogenicity tests emphasize contact frequency and long-term effects faced by the compounds under investigation. GA, TQ, Fisetin, and CA were not carcinogenic in the FDA Carcinogenicity Male Rat Non vs. Carc (v3.1) test. Only Betanidin and Ebselen were not carcinogenic in the FDA Carcinogenicity Male Rat Single vs. Mult (v3.1) test. GA and CA were not carcinogenic in the FDA Carcinogenicity Female Rat Non vs. Carc (v3.1) test. GA, Betanidin, CA, Fisetin, Betanin, and Ebselen were not carcinogenic in the FDA Carcinogenicity Female Rat Single vs. Mult (v3.1) test. GA, Betanidin, Fisetin, TQ, and Ebselen were not carcinogenic in the FDA Carcinogenicity Male Mouse Non vs. Carc (v3.1) test. GA, Betanidin, TQ, CA, Fisetin, Betanin, and Ebselen were not carcinogenic in the FDA Carcinogenicity Male Mouse Single vs. Mult (v3.1) test. Only GA, Fisetin, and TQ were not carcinogenic in FDA Carcinogenicity Female Mouse Non vs. Carc (v3.1) test and Only GA, Fisetin, and Ebselen were not carcinogenic in FDA Carcinogenicity Female Mouse Single vs. Mult (v3.1) test.

Developmental Toxicity Potential indicates mutagenic characteristics during development that can restrict their use in the pregnancy. Only GA, TQ, and CA were safe. Skin Irritation test (v6.1) showed only GA, Fisetin, Betanin, and Ebselen do not irritate the skin. Skin Sensitization examination revealed all of the compounds that cause skin allergies in Sensitization NEG v SENS (v6.1) test and GA, TQ, CA, Betanin, and Ebselen through Skin Sensitization MLD/MOD v SEV (v6.1) test do not cause skin allergies. Ocular Irritancy test also showed GA, Betanidin, CA, Fisetin, Betanin, and Ebselen do not cause ocular irritation in Ocular Irritancy SEV vs. MOD (v5.1) test. In Ocular Irritancy MLD vs. NON (v5.1) test also GA, Fisetin, and Betanin did not cause ocular irritation and in the Ocular Irritancy, SEV/MOD vs. MLD/NON (v5.1) test only Fisetin and Ebselen did not result in ocular irritation. In the Aerobic Biodegradability test, Fisetin, Betanidin, TQ, and Ebselen were resistant to the effects of biodegradation. We concluded using TOPKAT and ADMET properties, GA, CA, and Betanin had the lowest toxicity and side effects.

## 4 Discussion

SUMO activation and deconjugation from targets are carried out by SENPs. It is difficult to study this posttranslational modification because of the lack of reagents to obstruct the removal of SUMO from targets (Albrow et al., 2011). SENP1 and SENP2 have equal substrate specificity (Cheng et al., 2007). SENP2 is a nuclear envelope-related protease and when upregulated, it possesses activity like SENP1 (Cheng et al., 2004). Cobenas-Potts *et al.* found SENP1 and SENP2 are situated to change spatial and temporal regulation of SUMOylation via unequaled assemblies with kinetochores, centrosomes, and spindle microtubules. SENP1 or SENP2 play roles in temporal and spatial regulation of SUMOylation in mitosis and alteration of their expression cause to failings in chromosome congression in prometaphase or sister chromatid separation at metaphase. *SENP2* upregulation induces a defect in chromosomal congression and pertains to its accurate kinetochore pointing (Cubeñas-Potts et al., 2013). In addition to, *SENP2* upregulation in murine hearts induced cardiac dysfunction and congenital heart defects (Kim et al., 2012). Inhibition of Akt deSUMOylation by SENP2 can raise cardiomyocyte proliferation and angiogenesis to improve cardiac function after myocardial infarction (Chen et al., 2021).

In this study, we examined GA, CA, TQ, Betanin, Betanidin, Ebselen, and Fisetin. Docking and simulation results showed that among these ligands, Betanin is the most stable compound in the binding to SENP2. Betanin showed the lowest RMSD value (0.21 nm). Betanin also constituted four to seven hydrogen bonds and had the highest potential energy (-656,986 kJ/mol) even than Ebselen as inhibitor of SENP2. Betanin also represented the highest MD binding energy (-328.872 kJ/mol) after Betanidin. Betanin with hydrogen bonds and hydrophobic bonds also formed a powerful complex with the active site of SENP2. Betanin also showed a low distance with SENP2 even than Ebselen. This *in silico* finding is consistent with the results of *in vivo* and *in vitro* studies. Betanin presents in *Beta vulgaris*. Betanin is a principal pigment and an active phytochemical of beetroot (Gliszczyńska-Świgło et al., 2006) that has the properties of anti-proliferative, antioxidant, free radical-scavenging, anti-inflammatory, and pro-apoptotic. Betanin through the activation of Nrf2 may induce the expression of phase II detoxifying enzymes in human non-tumor liver cells. Betanin with the activation of Nrf2 led to increased GSTM, GSTP, GSTT, and NQO1 mRNA and protein levels and GST and NQO1 activities in the THLE-2 cells. Betanin has significantly enhanced the mRNA and protein levels of p53 in the THLE-2 cells (Krajka-Kuźniak et al., 2013). Betanin induced apoptosis and inhibited carcinogenesis in esophageal (Lechner et al., 2010), myeloid leukemia (Sreekanth et al., 2007), skin, and lung (Kapadia et al., 1996). The different scientists reported the interaction of molecular docking of various Betalain compounds including Betanin against NS2/NS3 protease (ul Qamar et al., 2014), and Betanidin against Nrf2 activator (Gacesa et al., 2015). In another study, the interaction of Betalain to LOX1^1^ and COX2^2^ was reported (Vidal et al., 2014). These studies are representative of the anti-virus, anti-oxidative and anti-inflammatory properties of Betalain.

In the previous studies, we showed GA can inhibit SENP1 and can be explored as a drug for cancer treatment (Taghvaei et al., 2021b) and Bethanidine with SENP1 inhibition can be a suitable candidate against cardiovascular diseases (Taghvaei et al., 2021a). Kumar *et al.* introduced 1, 2, 5-Oxadiazoles as SENP2 inhibitors (Kumar et al., 2014). Kumar *et al* docked Namiki-shoji small molecule library with ∼4 million commercially available compounds. We also docked 1, 2, 5-Oxadiazoles with IC50 < 10 µM which were displayed in Table 6. We observed our compounds have the lowest binding energy very close to 1, 2, 5-Oxadiazoles (Kumar et al., 2014).

**TABLE 6:** molecular docking of 1, 2, 5-Oxadiazoles (from virtual screening of Namiki-shoji small molecule library with SENP2, Kumar, *et al.*) with IC50 < 10µM with SENP2.

Number	Name	IC50 (µM)	Lowest binding free energy (kcal/mol)
1	52	6.8	−6.68
2	59	7.6	−5.99
3	68	8.7	−5.83
4	69	5.9	−5.54
5	78	9.4	−4.83
6	91	7.7	−5.51
7	98	7.8	−5.39
8	101	7.5	−5.44
9	102	6.3	−5.22
10	106	8.2	−5.64
11	107	6.3	−5.63
12	108	6.2	−5.68
13	109	9.0	−5.44
14	111	7.0	−5.68
15	115	4.5	−4.02
16	117	3.7	−5.20
17	118	6.8	−5.25
18	119	6.3	−10.57
19	120	5.0	−5.06
20	121	4.4	−5.12
21	122	4.0	−5.24
22	123	5.2	−4.53
23	124	5.9	+1.07

We hope Betanin is useful in the treatment of various and complex diseases. Using natural compounds, side effects can be diminished and the cost of drugs can be decreased. Our results propose that SENP2 may be an attractive target for the design of new targets for cardiac regeneration in the future. The method used to identify and introduce higher binding power compounds to SENP2 as a potential therapeutic target is an effective way of identifying novel inhibitor compounds.

It is expected that this study will demonstrate the potential of Betanin for use in laboratory studies against SENP2 in heart defects and cardiac dysfunction which initially studies have demonstrated the potentiality of this drug-like compound.

## Data Availability

The datasets presented in this study can be found in online repositories. The names of the repository/repositories and accession number(s) can be found in the article/Supplementary Material.

## Footnotes

1 Lipoxygenase1.

2 Cyclooxygenase2.
