# Supplementary material for: Identification of Natural Products as SENP2 Inhibitors for Targeted Therapy in Heart Failure
Source: Front Pharmacol. 2022 Apr 1;13:817990. doi: 10.3389/fphar.2022.817990 (PMC9012495; doi:10.3389/fphar.2022.817990)
Supplement: Supplementary file 1 [file DataSheet1.docx]

Supplementary Material

***Identification of Natural Products as SENP2 Inhibitors for Targeted Therapy in Heart Failure***

The supplementary material contains 3 Tables and 2 Figures. Supplementary Table 1 is the number, codeID, and 2D structure of these natural products. Supplementary Table 2 is ADMET results in 2 Tables. Supplementary Table 3 is our TOPCAT results in 2 Tables. Supplementary Figure 1 is the interaction images of the compounds by LigPlot in molecular docking and Supplementary Figure 2 is the interaction images of the compounds by Discovery Studio in molecular dynamics simulation.

**Supplementary Table 1**: Number, codeID with 2D structure of compounds

| **3D structure** | **CodeID** | **Compound Name** | **number** |
| --- | --- | --- | --- |
|  | ZINC1504 | Gallic acid | 1 |
|  | ZINC58172 | Caffeic acid | 2 |
|  | ZINC164367 | Thymoquinone | 3 |
|  | ZINC62592125 | Betanin | 4 |
|  | ZINC1531169 | Betanidin | 5 |
|  | ZINC39111 | Fisetin | 6 |
|  | Chemspider ID:3082 | Ebselen | 7 |

**Supplementary Table 2:** ADMET results: The results of the software are on line ADMETSAR (compounds: ZINC1531169, ZINC39111, ZINC164367**)**

AdmetSAR on line software for ADMET prediction, N.I.=Non-Inhibitor., I.= Inhibitor, N.S.= Non-Substrate, S.= Substrate, N.R.= Non-required

|  |  |  | **ZINC1531169** |  | **ZINC39111** |  | **ZINC164367** |
| --- | --- | --- | --- | --- | --- | --- | --- |
| Drug |  | Results | Probability |  |  | Results | Probability |
| Absorption | Blood Brain Barrier | BBB^-^ | 0.7743 | BBB+ | 0.5116 | BBB+ | 0.8138 |
|  | Human Intestinal | HIA- | 0.8496 | HIA+ | 0.9833 | HIA+ | 1.0000 |
|  | Absorption |  |  |  |  |  |  |
|  | Caco-2 Permeability | Caco2- | 0.6338 | Caco2- | 0.8367 | Caco2+ | 0.8019 |
|  | P-glycoprotein S. | S | 0.7807 | S | 0.5510 | NS | 0.7029 |
|  | P-glycoprotein I. | NI | 0.8813 | NI | 0.9018 | I | 0.6315 |
|  |  | NI | 0.9438 | NI | 0.8259 | NI | 0.9359 |
|  | Renal  Organic Cation  Transporter | NI | 0.8110 | NI | 0.9242 | NI | 0.8733 |
|  |  |  | | | | | |
|  |  |  |  |  |  |  |  |
|  | Subcellular Location | Mitochondria | 0.5551 | Mitochondria | 0.7742 | Mitochondria | 0.8182 |
| Distribution | CYP450 2C9 S | NS | 0.7625 | NS | 0.8088 | NS | 0.8366 |
| Metabolism | CYP450 2D6 S. | NS | 0.8077 | NS | 0.9110 | NS | 0.8381 |
|  | CYP450 3A4 S. | S | 0.5054 | NS | 0.6630 | NS | 0.5000 |
|  | CYP450 1A2 I. | NI | 0.5430 | I | 0.9249 | NI | 0.7977 |
|  | CYP450 2C9 I. | NI | 0.6626 | I | 0.8949 | NI | 0.8209 |
|  | CYP450 2D6 I. | NI | 0.6850 | NI | 0.9230 | NI | 0.8339 |
|  | CYP450 2C19 I. | NI | 0.6987 | NI | 0.6965 | NI | 0.7249 |
|  | CYP450 3A4 I. | NI | 0.9236 | NI | 0.7054 | NI | 0.9062 |
|  | CYP Inhibitory | Low | 0.8432 | Low | 0.5409 | Low | 0.6762 |
|  | Promiscuity |  |  |  |  |  |  |
| Excretion | Human Ether-a- | Weak | 0.9480 | Weak | 0.9774 | Weak | 0.8588 |
| Toxicity | go-go-Related Gene | Inhibitor |  | Inhibitor |  | Inhibitor |  |
|  | inhibition | NI | 0.8089 | NI | 0.8374 | NI | 0.9639 |
|  | AMES Toxicity | Non- AMES | 0.5000 | Non- AMES | 0.5905 | Non- AMES | 0.9336 |
|  |  | toxic |  | toxic |  | toxic |  |
|  | Carcinogens | Non- carcinogens | 0.9219 | Non- carcinogens | 0.9390 | Non- carcinogens | 0.7160 |
|  | Fish Toxicity | High | 0.9917 | High | 0.9766 | High | 0.6295 |
|  | (FHMT) |  |  |  |  |  |  |
|  | Tetrahymena | High | 0.9125 | High | 0.9971 | High | 0.8015 |
|  | Pyriformis Toxicity |  |  |  |  |  |  |
|  | (TPT) |  |  |  |  |  |  |
|  | Honey Bee Toxicity | Low | 0.7121 | High | 0.6228 | High | 0.8796 |
|  | (HBT) |  |  |  |  |  |  |
|  | Biodegradation | Not ready | 0.9392 | Not ready | 0.8902 | Not ready | 0.5353 |
|  |  | biodegradable |  | biodegradable |  | biodegradable |  |
|  | Acute Oral Toxicity | III | 0.5553 | II | 0.7187 | II | 0.6763 |
|  | Carcinogenicity | Non-R | 0.5292 | Non-R | 0.5926 | Non-R | 0.6030 |
|  | (three-class) |  |  |  |  |  |  |
| Absorption | Aqueous solubility, | -2.9602 | LopS | -3.0804 | LopS | -2.0061 | LopS |
|  | LogS |  |  |  |  |  |  |
|  | Caco-2 | -0.0941 | LogPapp, | 0.4029 | LogPapp, | 1.9554 | LogPapp, |
|  | Permeability, |  | cm/s |  | cm/s |  | cm/s |
|  | LogPapp, |  |  |  |  |  |  |
|  | cm/s |  |  |  |  |  |  |
| Toxicity | Rat Acute Toxicity | 2.5285 | LD50, mol/kg | 3.1831 | LD50, mol/kg | 2.6165 | LD50, mol/kg |
|  | LD50, mol/kg |  |  |  |  |  |  |
|  | Fish Toxicity pLC50  mg/L | 0.7864 | pLC50,mg/L | 0.2432 | pLC50,mg/L | 1.0093 | pLC50. |
|  | Tetrahymena  Pyriformis Toxicity  pIGC50, ug/L | 0.5165 | pIGC50, ug/L | 0.5944 | pIGC50, ug/L | -0.0239 | pIGC50, ug/L |

**Supplementary Table 2 Continued:** ADMET results: The results of the software are on line ADMETSAR

(Compounds: ZINC1504, ZINC58172, ZINC62592125, and Chemspider ID:3082)

AdmetSAR on line software for ADMET prediction, N.I.=Non-Inhibitor., I.= Inhibitor, N.S.= Non-Substrate, S.= Substrate, N.R.= Non-required

|  |  |  | **ZINC1504** |  | **ZINC58172** |  | **ZINC62592125** |  | **Chemspider 3082** |
| --- | --- | --- | --- | --- | --- | --- | --- | --- | --- |
| Drug |  | Results | Probability | Results | Probability | Results | Probability | Results |  |
| Absorption | Blood Brain Barrier | BBB- | 0.6225 | BBB- | 0.6322 | BBB- | 0.7744 | BBB+ | 0.9979 |
|  | Human Intestinal | HIA+ | 0.8051 | HIA+ | 0.9392 | HIA- | 0.9530 | HIA+ | 1.0000 |
|  | Absorption |  |  |  |  |  |  |  | 0.6654 |
|  | Caco-2 Permeability | Caco2- | 0.5611 | Caco2+ | 0.5693 | Caco2- | 0.6622 | Caco2- | 0.6850 |
|  | P-glycoprotein S. | NS | 0.6639 | NS | 0.6451 | S | 0.7945 | NS | 0.8764 |
|  | P-glycoprotein I. | NI | 0.9825 | NI | 0.9738 | NI | 0.8393 | NI | 0.6850 |
|  |  | NI | 0.9940 | NI | 0.9935 | NI | 0.8355 | NI | 0.6060 |
|  | Renal  Organic Cation  Transporter | NI | 0.9415 | NI | 0.9387 | NI | 0.7801 | NI | 0.8220 |
|  |  |  | | | | | | | |
|  |  |  |  |  |  |  |  |  |  |
|  | Subcellular Location | Mitochondria | 0.7287 | Mitochondria | 0.8027 | Nucleus | 0. 4320 | Mitochondria | 0.7292 |
| Distribution | CYP450 2C9 S | NS | 0.8198 | NS | 0.8014 | NS | 0.7840 | NS | 0.7237 |
| Metabolism | CYP450 2D6 S. | NS | 0.9193 | NS | 0.9136 | NS | 0.8144 | NS | 0.8359 |
|  | CYP450 3A4 S. | NS | 0.7265 | NS | 0.7046 | S | 0.5111 | S | 0.6381 |
|  | CYP450 1A2 I. | NI | 0.9274 | NI | 0.9046 | NI | 0.6095 | I | 0.9106 |
|  | CYP450 2C9 I. | NI | 0.9363 | NI | 0.9071 | NI | 0. 7692 | I | 0.8949 |
|  | CYP450 2D6 I. | NI | 0.9693 | NI | 0.9525 | NI | 0. 8291 | I | 0.8931 |
|  | CYP450 2C19 I. | NI | 0.9782 | NI | 0.9367 | NI | 0.8021 | I | 0.8994 |
|  | CYP450 3A4 I. | NI | 0.8427 | NI | 0.8869 | NI | 0.8947 | I | 0.5000 |
|  | CYP Inhibitory | Low | 0.9318 | Low | 0.9007 | Low | 0. 8348 | High | 0.8045 |
|  | Promiscuity |  |  |  |  |  |  |  |  |
| Excretion | Human Ether-a- | Weak | 0.9821 | Weak | 0.9763 | Weak | 0.9008 | Weak | 0.9718 |
| Toxicity | go-go-Related Gene | Inhibitor |  | inhibitor |  | inhibitor |  | inhibitor |  |
|  | inhibition | NI | 0.9599 | NI | 0.9548 | NI | 0.7159 | NI | 0.7381 |
|  | AMES Toxicity | Non- AMES toxic | 0.9146 | Non- AMES toxic | 0.9132 | Non- AMES toxic | 0.5822 | Non- AMES toxic | 0.7082 |
|  | Carcinogens | Non- carcinogens | 0.9177 | Non-carcinogens | 0.9183 | Non-carcinogens | 0.9207 | Non-carcinogens | 0.9122 |
|  | Fish Toxicity | High | 0.9072 | High | 0.9801 | High | 0.9672 | High | 0.9484 |
|  | (FHMT) |  |  |  |  |  |  |  |  |
|  | Tetrahymena | High | 0.8663 | High | 0.9580 | High | 0.9524 | High | 0.8824 |
|  | Pyriformis Toxicity |  |  |  |  |  |  |  |  |
|  | (TPT) |  |  |  |  |  |  |  |  |
|  | Honey Bee Toxicity | High | 0.6665 | High | 0.7041 | High | 0.6267 | Low | 0.7754 |
|  | (HBT) |  |  |  |  |  |  |  |  |
|  | Biodegradation | Ready biodegradable | 0.8936 | Ready biodegradable | 0.8012 | Not-ready biodegradable | 0.8719 | Not-ready biodegradable | 0.9883 |
|  | Acute Oral Toxicity | III | 0.6904 | IV | 0.5588 | III | 0.6038 | III | 0.6927 |
|  | Carcinogenicity | Non-R | 0.7405 | Non-R | 0.5848 | Non-R | 0.5301 | Warning | 0.3954 |
|  | (three-class) |  |  |  |  |  |  |  |  |
| Absorption | Aqueous solubility, | -1.0973 | LopS | -1.6939 | LopS | -2.5688 | LopS | -3.4907 | LopS |
|  | LogS |  |  |  |  |  |  |  |  |
|  | Caco-2 | 0.0595 | LogPap | 0.3485 | LogPapp | -0.4282 | LogPapp, | 1.6360 | LogPapp, |
|  | Permeability, |  | p, cm/s |  | cm/s |  | cm/s |  | cm/s |
|  | LogPapp, |  |  |  |  |  |  |  |  |
|  | cm/s |  |  |  |  |  |  |  |  |
| Toxicity | Rat Acute Toxicity | 1.8670 | LD50, mol/kg | 1.4041 | LD50,mol/kg | 2.4833 | LD50,mol/kg | 2.2143 | LD50,mol/kg |
|  | LD50, mol/kg |  |  |  |  |  |  |  |  |
|  | Fish Toxicity,  pLC50. | 1.3371 | pLC50. mg/L | 0.7921 | pLC50. mg/L | 0.8206 | pLC50. mg/L | 0.8807 | pLC50. mg/L |
|  | Tetrahymena  Pyriformis Toxicity,  pIGC50, ug/L | -0.6608 | pIGC50, ug/L | -0.2480 | pIGC50, ug/L | 0.5557 | pIGC50, ug/L | 0.6005 | pIGC50, ug/L |

**Supplementary Table 3:** TOPKAT results for compounds, results from the TOPKAT module in the software Discovery Studio 2.5, (Compounds: ZINC1504, ZINC1531169, and ZINC164367).

| **ZINC164367** | **ZINC1531169** | **ZINC1504** | **Test** |
| --- | --- | --- | --- |
| 0.000 | 0.000 | 0.000 | Ames Mutagenicity (v3.1) |
| 0.973 | 0.901 | 0.000 | NTP Carcinogenicity Call (Male Rat) (v3.2) |
| 0.000 | 0.000 | 0.000 | NTP Carcinogenicity Call (Female Rat) (v3.2) |
| 0.000 | 0.000 | 0.000 | NTP Carcinogenicity Call (Male Mouse) (v3.2) |
| 0.045 | 0.997 | 0.000 | NTP Carcinogenicity Call (Female Mouse) (v3.2) |
| 0.000 | 1.000 | 0.000 | FDA Carcinogenicity Male Rat Non vs Carc (v3.1) |
| 0.388 | 0.000 | 1.000 | FDA Carcinogenicity Male Rat Single vs Mult (v3.1) |
| 0.433 | 1.000 | 0.000 | FDA Carcinogenicity Female Rat Non vs Carc (v3.1) |
| 0.999 | 0.000 | 0.222 | FDA Carcinogenicity Female Rat Single vs Mult (v3.1) |
| 0.037 | 0.004 | 0.266 | FDA Carcinogenicity Male Mouse Non vs Carc (v3.1) |
| 0.000 | 0.000 | 0.000 | FDA Carcinogenicity Male Mouse Single vs Mult (v3.1) |
| 0.000 | 1.000 | 0.004 | FDA Carcinogenicity Female Mouse Non vs Carc (v3.1) |
| 1.000 | 1.000 | 0.008 | FDA Carcinogenicity Female Mouse Single vs Mult (v3.1) |
| 0.000 | 1.000 | 0.000 | Weight of Evidence Carcinogenicity Call (v5.1) |
| 0.034 | 0.999 | 0.129 | Developmental Toxicity Potential (DTP) (v3.1) |
| 131.9 | 685.9 | 5 | Rat Oral LD50 (v3.1) (mg/kg) |
| 312.6 | 33.8 | 49.6 | Rat Maximum Tolerated Dose - Feed/Water (v6.1) (mg/kg) |
| 312.6 | 33.8 | 49.6 | Rat Maximum Tolerated Dose - Gavage (v6.1) (mg/kg) |
| 6.1 | 10 | 689.2 | Rat Inhalational LC50 (v6.1) (mg/m3/H) |
| 487.4 | 10 | 1.4 | Chronic LOAEL (v3.1) (mg/kg) |
| 1.000 | 1.000 | 0.000 | Skin Irritation (v6.1) |
| 0.878 | 1.000 | 1.000 | Skin Sensitization NEG v SENS (v6.1) |
| 0.000 | 1.000 | 0.19 | Skin Sensitization MLD/MOD v SEV (v6.1) |
| 0.973 | 1.000 | 1.000 | Ocular Irritancy SEV/MOD vs MLD/NON (v5.1) |
| 0.994 | 0.000 | 0.000 | Ocular Irritancy SEV vs MOD (v5.1) |
| 0.990 | 1.000 | 0.013 | Ocular Irritancy MLD vs NON (v5.1 |
| 0.144 | 0.000 | 1.000 | Aerobic Biodegradability (v6.1) |
| Error | 43.3 | - | Fathead Minnow LC50 (v3.2) (mg/l) |
| 3.9 | 5.8 | 7.4 | Daphnia EC50 (v3.1) (mg/l) |
| 2.550 | 2.853 | 0.968 | LogP (v3.1) |

**Supplementary Table 3 Continued:** TOPKAT results for compounds, results from the TOPKAT module in the software Discovery Studio 2.5, compounds: ZINC58172, ZINC62592125, ZINC39111, and Chemspider 3082)

| **ZINC39111** | **ZINC62592125** | **ZINC58172** | **ChemSpider 3082** | **Test** |
| --- | --- | --- | --- | --- |
| 1.000 | 0.986 | 0.964 | 0.996 | Ames Mutagenicity (v3.1) |
| 0.994 | 0.008 | 0.007 | 1.000 | NTP Carcinogenicity Call (Male Rat) (v3.2) |
| 1.000 | 0.000 | 0.993 | 0.997 | NTP Carcinogenicity Call (Female Rat) (v3.2) |
| 1.000 | 0.123 | 0.027 | 0.000 | NTP Carcinogenicity Call (Male Mouse) (v3.2) |
| 0.000 | 0.007 | 0.010 | 0.002 | NTP Carcinogenicity Call (Female Mouse) (v3.2) |
| 0.001 | 0.531 | 0.025 | 1.000 | FDA Carcinogenicity Male Rat Non vs Carc (v3.1) |
| 1.000 | 1.000 | 1.000 | 0.000 | FDA Carcinogenicity Male Rat Single vs Mult (v3.1) |
| 1.000 | 0.308 | 0.068 | 0.999 | FDA Carcinogenicity Female Rat Non vs Carc (v3.1) |
| 0.000 | 0.000 | 0.000 | 0.000 | FDA Carcinogenicity Female Rat Single vs Mult (v3.1) |
| 0.072 | 0.363 | 0.487 | 0.001 | FDA Carcinogenicity Male Mouse Non vs Carc (v3.1) |
| 0.000 | 0.054 | 0.117 | 0.000 | FDA Carcinogenicity Male Mouse Single vs Mult (v3.1) |
| 0.000 | 0.998 | 0.999 | 1.000 | FDA Carcinogenicity Female Mouse Non vs Carc (v3.1) |
| 0.016 | 0.694 | 0.525 | 0.002 | FDA Carcinogenicity Female Mouse Single vs Mult (v3.1) |
| 0.000 | 0.000 | 0.003 | 0.288 | Weight of Evidence Carcinogenicity Call (v5.1) |
| 0.998 | 0.032 | 0.036 | 1.000 | Developmental Toxicity Potential (DTP) (v3.1) |
| 156.3 | 1.3 | 1.1 | 6.2 | Rat Oral LD50 (v3.1) (mg/kg) |
| 117.7 | 213.2 | 227.5 | 130.9 | Rat Maximum Tolerated Dose - Feed/Water (v6.1) (mg/kg) |
| 325.8 | 213.2 | 227.5 | 362.3 | Rat Maximum Tolerated Dose - Gavage (v6.1) (mg/kg) |
| 10 | 2.8 | 2.5 | 22.7 | Rat Inhalational LC50 (v6.1) (mg/m3/H) |
| 270.8 | 516.3 | 137.3 | 73.9 | Chronic LOAEL (v3.1) (mg/kg) |
| 0.000 | 0.000 | 0.997 | 0.000 | Skin Irritation (v6.1) |
| 1.000 | 0.992 | 0.992 | 0.992 | Skin Sensitization NEG v SENS (v6.1) |
| 0.989 | 0.079 | 0.037 | 0.000 | Skin Sensitization MLD/MOD v SEV (v6.1) |
| 0.000 | 0.998 | 0.998 | 0.243 | Ocular Irritancy SEV/MOD vs MLD/NON (v5.1) |
| 0.000 | 0.000 | 0.003 | 0.000 | Ocular Irritancy SEV vs MOD (v5.1) |
| 0.000 | 0.000 | 0.019 | 1.000 | Ocular Irritancy MLD vs NON (v5.1 |
| 0.000 | 1.000 | 1.000 | 0.913 | Aerobic Biodegradability (v6.1) |
| 322.1 | 2.1 | 3.9 | 5.2 | Fathead Minnow LC50 (v3.2) (mg/l) |
| 1.245 | 220.1 | 20.9 | 9.9 | Daphnia EC50 (v3.1) (mg/l) |
| 1.8 | 1.609 | 1.222 | 3.862 | LogP (v3.1) |

**Supplementary Figure 1**: LigPlot figures of compounds in molecular docking

| Molecular docking Figure | Natural product name |
| --- | --- |
| 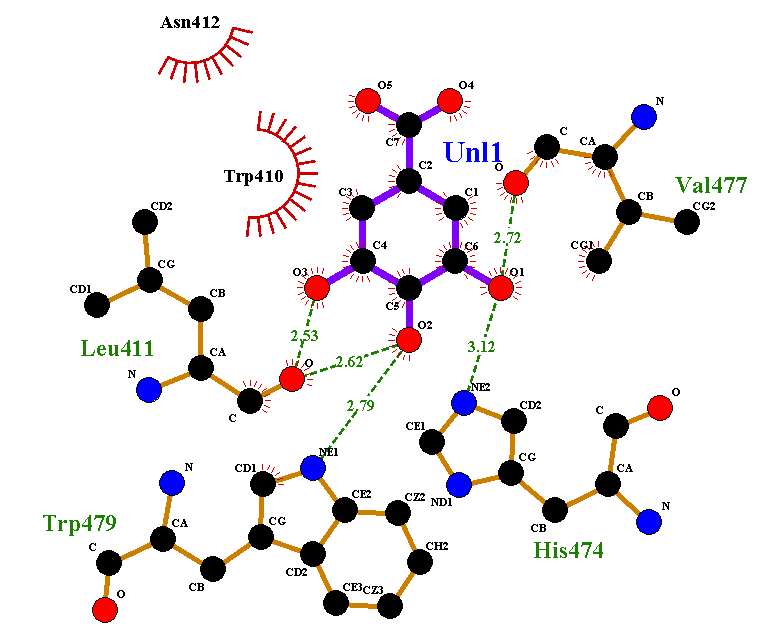 | Gallic Acid |
| 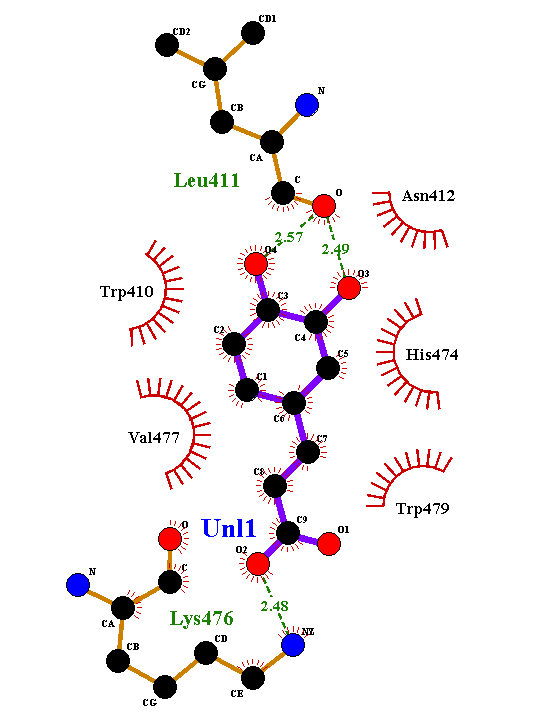 | Caffeic Acid |
| 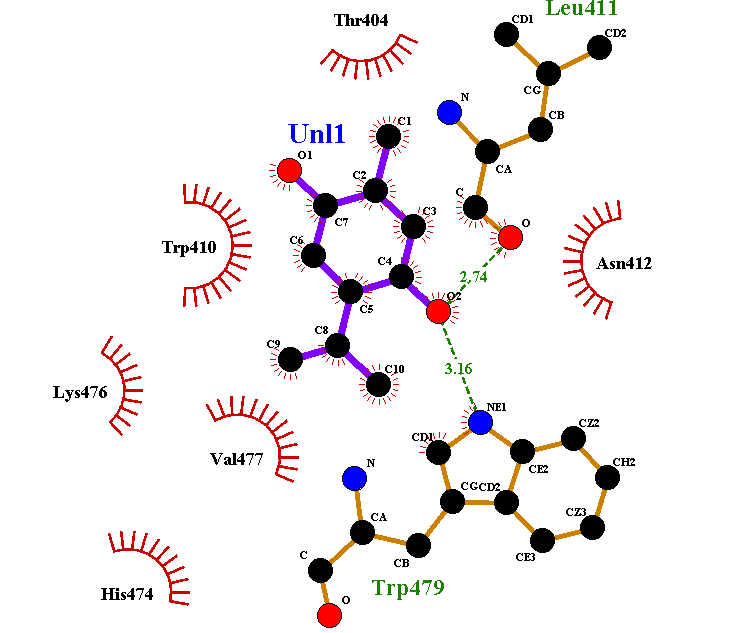 | Thymoquinone |
| 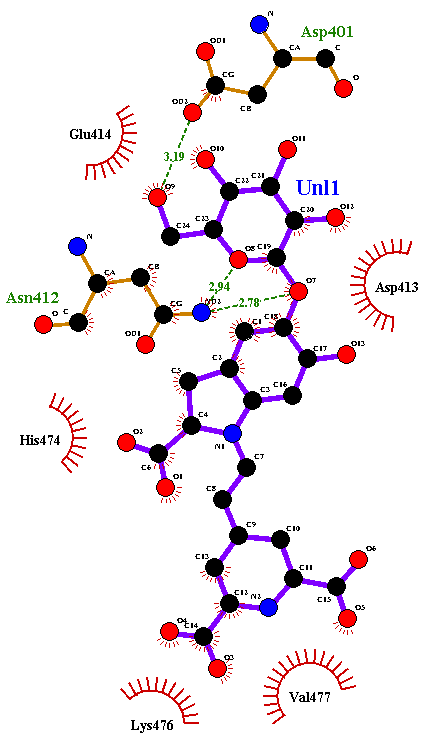 | Betanin |
| 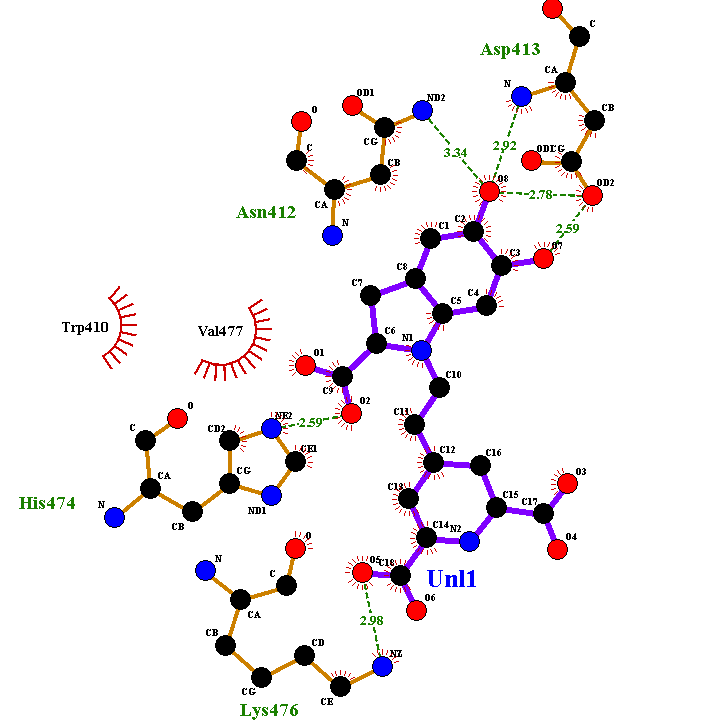 | Betanidin |
| 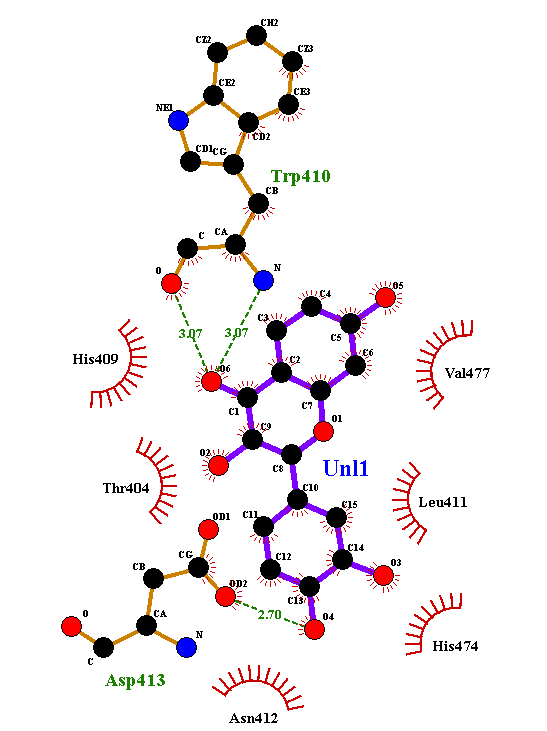 | Fisetin |
| 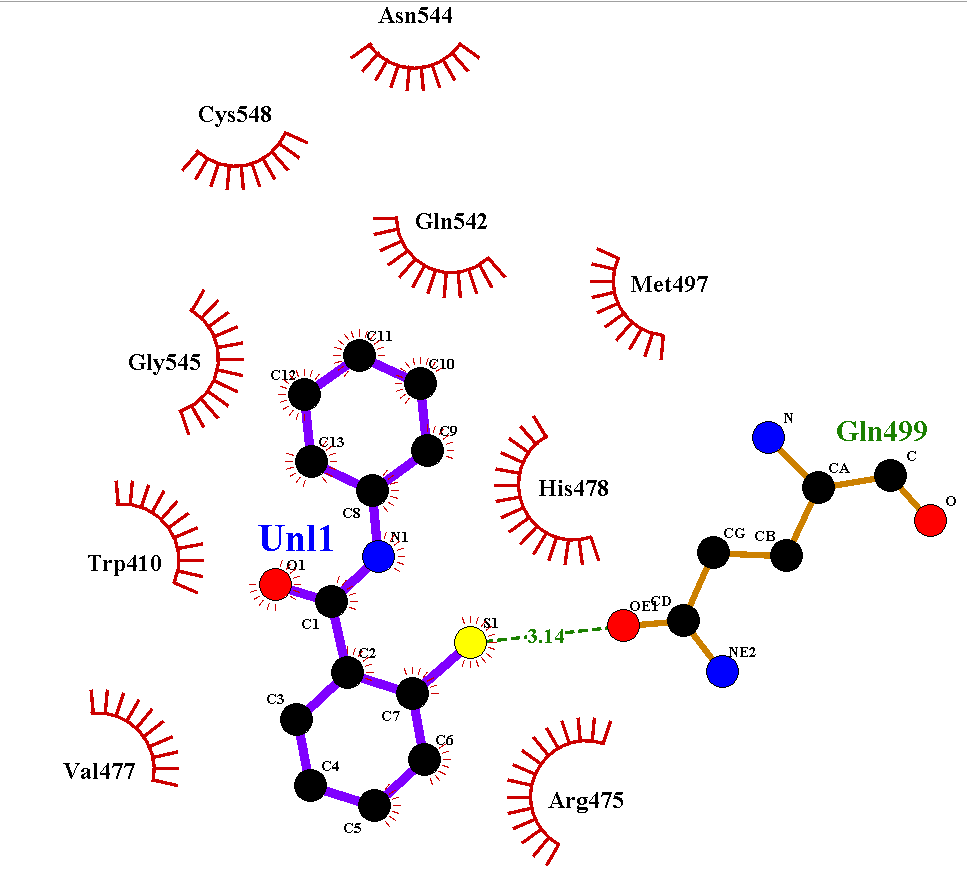 | Ebselen |
| 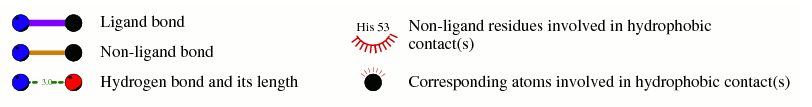 | |

**Supplementary Figure 2**: Discovery Studio figures of compounds in molecular dynamics simulation

| Natural product name | Molecular dynamic Figure |
| --- | --- |
| Gallic acid | 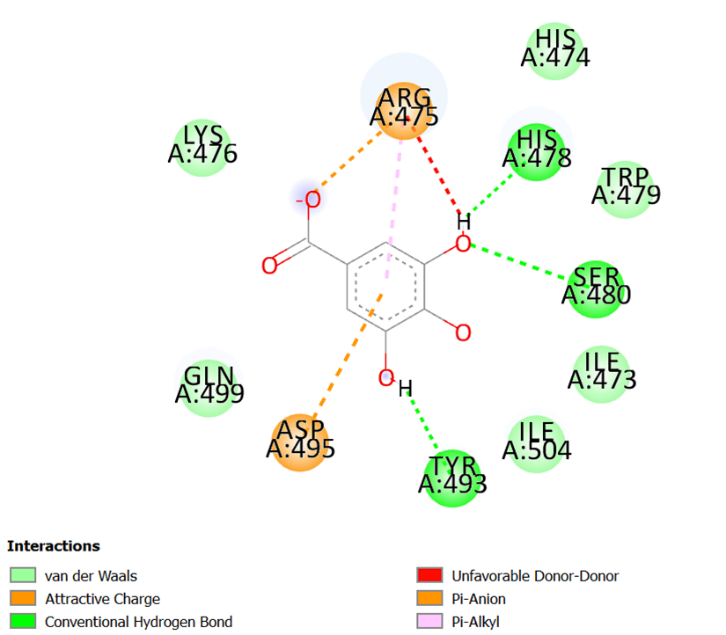 |
| Caffeic acid | 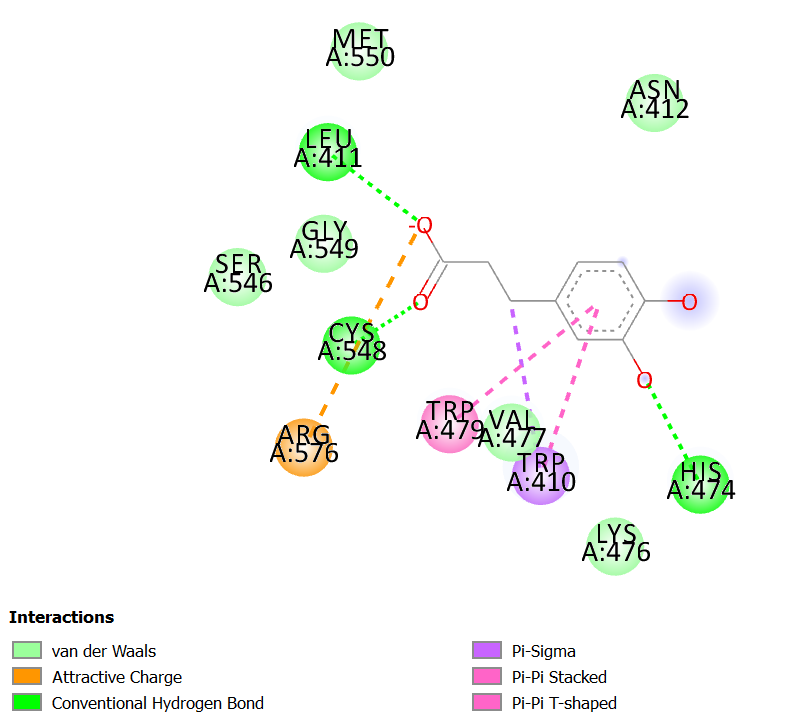 |
| Thymoquinone | 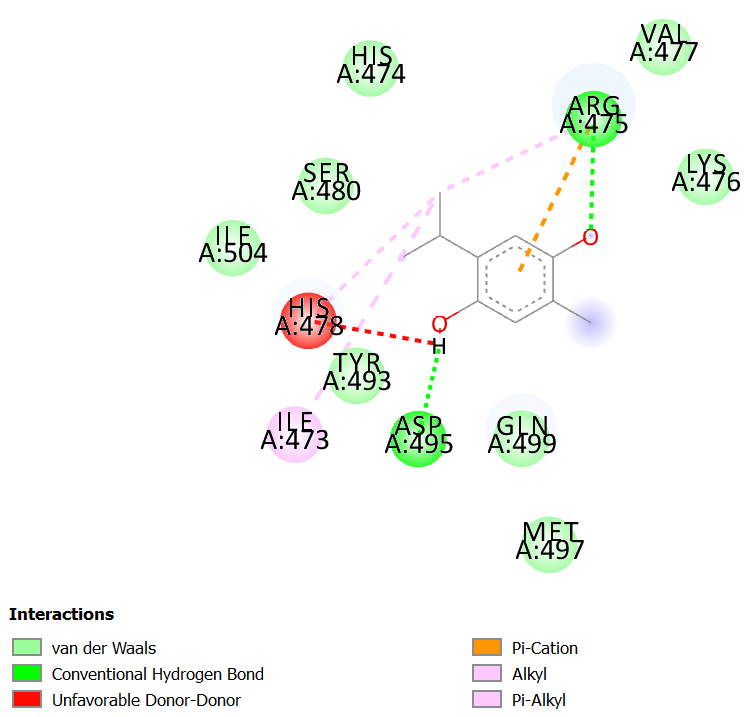 |
| Fisetin | 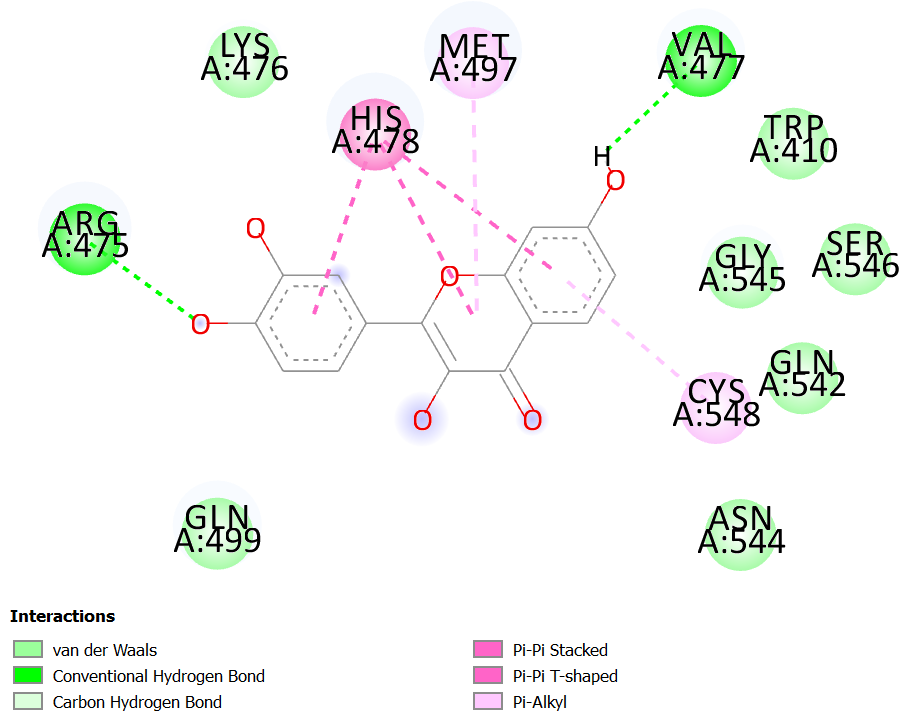 |
